# Dipeptidyl peptidase-IV inhibitory action of Calebin A: An *in silico* and *in vitro* analysis

**DOI:** 10.1016/j.jaim.2021.08.008

**Published:** 2021-10-29

**Authors:** Nehru Sai Suresh Chalichem, Srikanth Jupudi, Venkata Ramesh Yasam, Duraiswamy Basavan

**Affiliations:** a Department of Pharmacognosy and Phytochemistry, JSS College of Pharmacy (JSS Academy of Higher Education and Research, Mysuru, INDIA), Ooty, 643001, India; b Department of Pharmaceutical Chemistry, JSS College of Pharmacy (JSS Academy of Higher Education and Research, Mysuru, INDIA), Ooty, 643001, India; c Department of Pharmaceutics, JSS College of Pharmacy (JSS Academy of Higher Education and Research, Mysuru, INDIA), Ooty, 643001, India

**Keywords:** Alzheimer's disease, Calebin A, Dipeptidyl peptidase-IV, Induced fit docking, CD26 activity assay and *in silico* analysis

## Abstract

**Background:**

Dipeptidyl peptidase-IV (DPP-IV) inhibitors, the enhancers of incretin are used for the treatment of diabetes. The non-glycaemic actions of these drugs (under developmental stage) also proved that repurposing of these molecules may be advantageous for other few complicated disorders like cardiovascular diseases, Parkinson's disease, Alzheimer's disease, etc.

**Objective:**

The present study was aimed to investigate the DPP-IV inhibitory potential of Calebin-A, one of the constituents of *Curcuma longa*.

**Material and methods:**

The phytoconstituent was subjected for various *in silico* studies (using Schrödinger Suite) like, Docking analysis, molecular mechanics combined with generalized Born model and solvent accessibility method (MMGBSA) and Induced fit docking (IFD) after validating the protein using Ramachandran plot. Further, the protein-ligand complex was subjected to molecular dynamic simulation studies for 50 nanoseconds. And finally, the results were confirmed through enzyme inhibition study.

**Results:**

*Insilico* results revealed possible inhibitory binding interactions in the catalytic pocket (importantly Glu205, Glu206 and Tyr 662 etc.) and binding affinity in terms of glide g-score and MMGBSA dG bind values were found to be −6.2 kcal/mol and −98.721 kcal/mol. Further, the inhibitory action towards the enzyme was confirmed by an enzyme inhibition assay, in which it showed dose-dependent inhibition, with maximum % inhibition of 55.9 at 26.3 μM. From molecular dynamic studies (50 nanoseconds), it was understood that Calebin A was found to be stable for about 30 nanoseconds in maintaining inhibitory interactions.

**Conclusion:**

From the *in silico* and *in vitro* analysis, the current research emphasizes the consideration of Calebin A to be as a promising or lead compound for the treatment of several ailments where DPP-IV action is culprit.

## Graphical abstract

**Figure:**
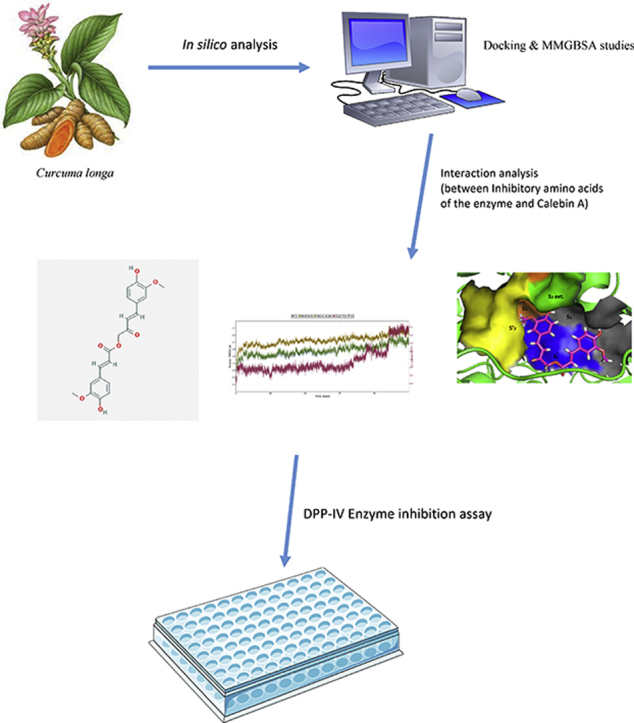

## 1 Introduction

Phytoconstituents are being used in drug discovery process for the exploration of lead candidates as they remain a source of new chemical moieties [1]. The rhizomes of *Curcuma longa* have numerous therapeutic applications and are widely used in traditional medicine for various ailments. In this context, our research group aimed to find the therapeutic importance of various active constituents of *C. longa,* one of the important Indian spice plants, via biological and chemical investigations. So far, great attention has been paid to pharmacological research of curcumin, but no significant work has been carried out on other active natural compounds like Calebin A (CA) [2]. In this scenario, we have carried out *in* *silico* and *in vitro* studies to find out the dipeptidyl peptidase-IV (DPP-IV) inhibitory activity of CA, one of the important constituents of *C. longa.*

In previous studies, anti-inflammatory, anti-cancer, and anti-obesity effects of CA were proved and moreover, it was observed that CA can ameliorate RANKL-induced osteoclastogenesis [[3], [4], [5], [6]]. On the other hand, latest advancements in the computational methods have opened new frontiers in the novel drug discovery and development process, especially in lead identification and optimization stages. Computational methods help us in understanding the drug binding affinity through molecular level interaction analysis that help to understand the stimulatory/inhibitory action of the molecules and to tackle the false positive leads.

Currently, DPP-IV inhibitors are being used for the treatment of Type 2 Diabetes Mellitus (T2DM). The DPP-IV, a serine protease was first revealed as glycylproline β-naphthylamidase [7]. It widely expresses on various cell types like epithelium of renal proximal tubules, intestine, corpus luteum, T_H_ cells and also on the subsets of macrophages and also as a soluble form in semen, plasma, and urine [8]. Crystallographic studies detailed about structure and amino acid arrangement of the enzyme. The active site of the enzyme lies in the large inner cavity which is formed by α/β hydrolase domain and β propeller domain [9,10]. The “side opening” that connects the inner cavity with the surrounding solvent, promotes the entry and exit of the ligands. Transmembrane part of the enzyme is hydrophobic in nature with 22 amino acids, cytoplasmic tail is short in nature with 6 amino acids, and remaining 738 amino acids constitutes extra-cellular domain [11,12].

The enzyme acts on several natural substrates like chemokines, cytokines, neuropeptides, circulating hormones, and bioactive peptides which acts as a regulatory switch for peptide hormonal metabolism and amino acid transport. This serine protease acts at proline or alanine at the N-terminal penultimate (P) position and promotes either activation or inactivation of the substrates. The non-glycaemic actions of these drugs also proved that repurposing of these molecules can be advantageous for other few complicated disorders like Alzheimer's disease for which our lab is currently working [[13], [14], [15]]. Hence, in this context we have carried out *in silico* studies viz*.,* Molecular mechanics combined with generalized Born model and solvent accessibility method (MMGB-SA), induced fit docking (IFD) and (simulation) dynamics for 50 nanoseconds (ns) to understand the possible behaviour of the molecule in the active site followed by enzyme inhibition assay to confirm the inhibitory action of the molecule.

## 2 Materials and methods

### 2.1 Molecular docking studies

X-ray crystal structure of human DPP-IV co-crystalized with Teneligliptin (PDB ID: 3VJK) was retrieved from the protein data bank and further prepared using protein preparation wizard (Epik v3.5, Schrödinger suite 2016-1) [16]. The initial protein structure is a dimer, where the redundant chain having similar binding sites was removed along with water molecules, refining bond orders and addition of hydrogens was done. Missing side-chains were included by using prime (v4.3), and then protonation and tautomeric states for acidic and basic residues were generated at neutral pH (7.0) [17]. Later, by maintaining the Root mean square deviation (RMSD) of crystallographic heavy atoms at 0.30 Å, enzyme/protein was stabilised by minimising the energy using molecular force field OPLS_2005 (Optimized Potentials for Liquid Simulations) [18]. Initially, stabilised protein was validated by using Ramachandran plot [19]. Ligprep module (v3.7) of Schrödinger suite 2016-1 was employed for the preparation of ligand. Later, the prepared ligands were processed for refining bond orders and addition of missed hydrogen atoms. Low energy conformers were generated and finally energy minimization was done by OPLS_2005 force field [18]. A grid box (X = 52.0 Å, Y = 64.9 Å, Z = 34.65 Å) was generated at the centroid of active site keeping the van der Waals scaling of 0.8 for the receptor with 0.15 as the partial charge cut-off. The generated low energy poses were docked using Glide v7.0 into the active site of DPP-IV (PDB ID: 3VJK) using extra precision mode (XP) [20] keeping other parameters default [21]. The best docking pose was selected based on glide g-score and glide energy values.

### 2.2 IFD and free energy calculation studies

In order to evaluate the conformational changes induced by binding of CA in the active site, IFD protocol of Schrödinger suite, which combines glide docking and prime refinement modules was employed to generate accurate binding poses [17]. The ligand to be docked (with high glide score) was selected and grid box was generated keeping the co–crystal ligand as centroid core. Ligand and receptor van der Waals radii scaling were kept at 0.50 Å retaining maximum 20 poses per ligand and refining residues within 5 Å radius of ligand pose. The best binding pose of CA in complex with protein was ranked based on IFD scores. The contributions of enthalpy and entropy related components towards binding of ligand–protein complex was evaluated using prime MMGB-SA (v4.3) approach that integrates Generalised-Born/Surface Area (GB/SA) continuum solvent model and OPLS_2005 force field [18,22]. Binding free energy calculations were performed for IFD generated best complex [23].

### 2.3 Molecular dynamics (MD) simulation

In order to study the inhibitory behaviour of CA at atomic level, the molecular interaction analysis was performed using Desmond module of Schrödinger 2016-1, LLC, New York, NY. The complex of CA with DPP-IV enzyme was solvated with TIP3P water model [24] in an orthorhombic periodic boundary conditions having dimensions of 10 Å buffer region between protein atoms and box edges. The solvated system was neutralized by adding 13 Na^+^ as counter ions consisting of approximately 71915 atoms and 20059 water molecules. Later, the system was minimized using default OPLS_2005 force field parameters [18]. The long range electrostatic interactions were calculated at a tolerance of 1e-09 using smooth particle mesh Ewald method [25] whereas, the short-range van der Waals and Coulomb interactions were calculated at cut-off radius of 9.0 Å. By using a time step of 2fs, a simulation was done for a period of 50 nano seconds (ns) by keeping temperature at 300 k and pressure at 1 bar with an isothermal-isobaric ensemble (NPT). Nose–Hoover chain thermostat [26] and Martyna-Tobias-Klein barostat [27] methods were ensembled at 100 and 200ps respectively. A multiple time-step algorithms RESPA (REference System Propagator Algorithm) was used at 2, 2, and 6 fs for bonded, short-range non-bonded and long range electrostatic forces respectively. For every 100ps, the data were collected and the obtained trajectories were subjected for further analysis. MD was also performed for apo enzyme and enzyme with cocrystal.

### 2.4 Chemicals used

DPP-IV inhibitor screening assay kit was obtained from Cayman Chemicals (Catalog no. 700210) and stored at −80 °C, DMSO (Sigma Aldrich).

### 2.5 Enzyme inhibition analysis

#### 2.5.1 CA solution preparation

CA was obtained as a gift sample from Sabinsa Corporation, USA (≥95.4% by HPLC). Stock solution of 1 mg/ml concentration was prepared by using DMSO. From the stock solution, various concentrations were prepared with sequential dilutions using DMSO.

#### 2.5.2 Enzyme inhibition assay

The assay procedure was followed as per the kit instructions. In brief, the fluorogenic substrate, Gly-Pro-Aminomethylcoumarin was used at 100 μM as an assay concentration. After the addition of test samples as per the instructions, plate was incubated at 37 °C for 30 min, and then fluorescence reading was taken (single reading) by keeping instrumental gain as optimum and excitation and emission wavelengths were set at 355 nm and 455 nm respectively by using microplate reader (Tecan Infinite 200 Pro). Three individual experiments (triplicates in each experiment) were performed. In each experiment, percentage inhibition of each concentration and IC_50_ was calculated and final IC_50_ was represented as average of three individual experiments. Results were analysed through regression analysis using GraphPad Prism 6.0 and IC_50_ was expressed as Mean ± SD.

## 3 Results

### 3.1 Docking results

**Fig. 1:**
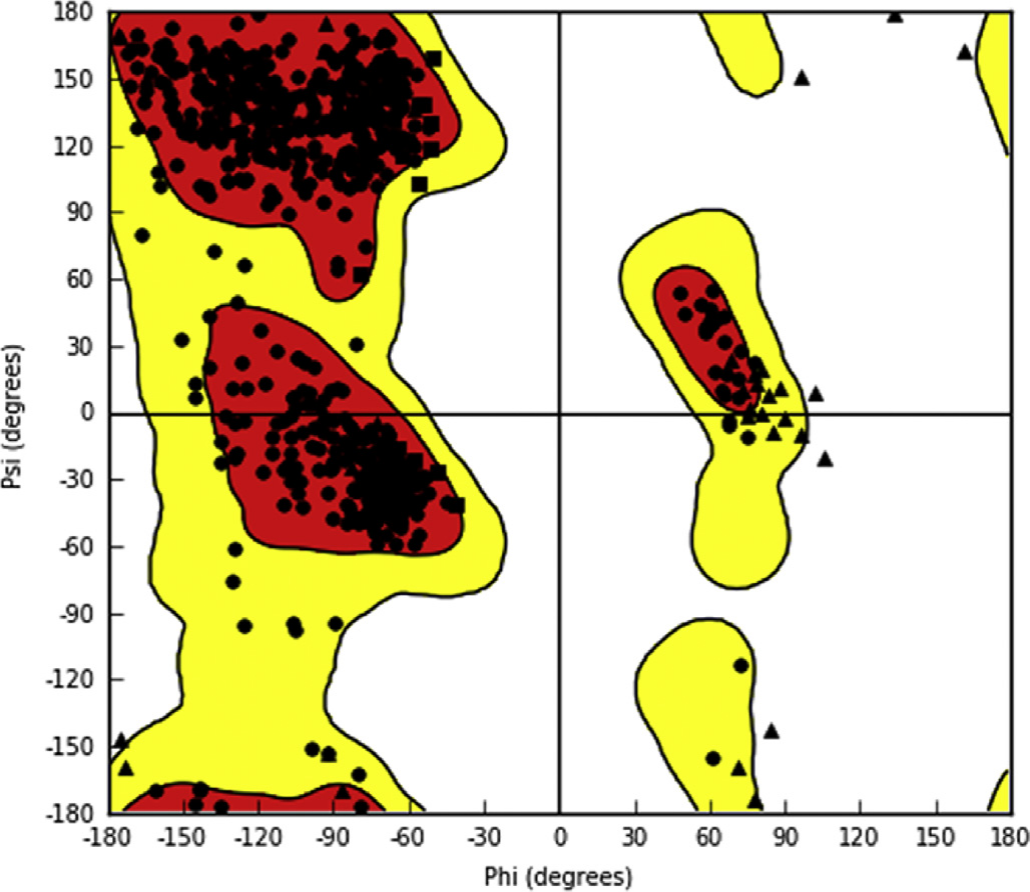
Ramachandran plot of prepared protein PDB ID: 3VJK.

**Fig. 2:**
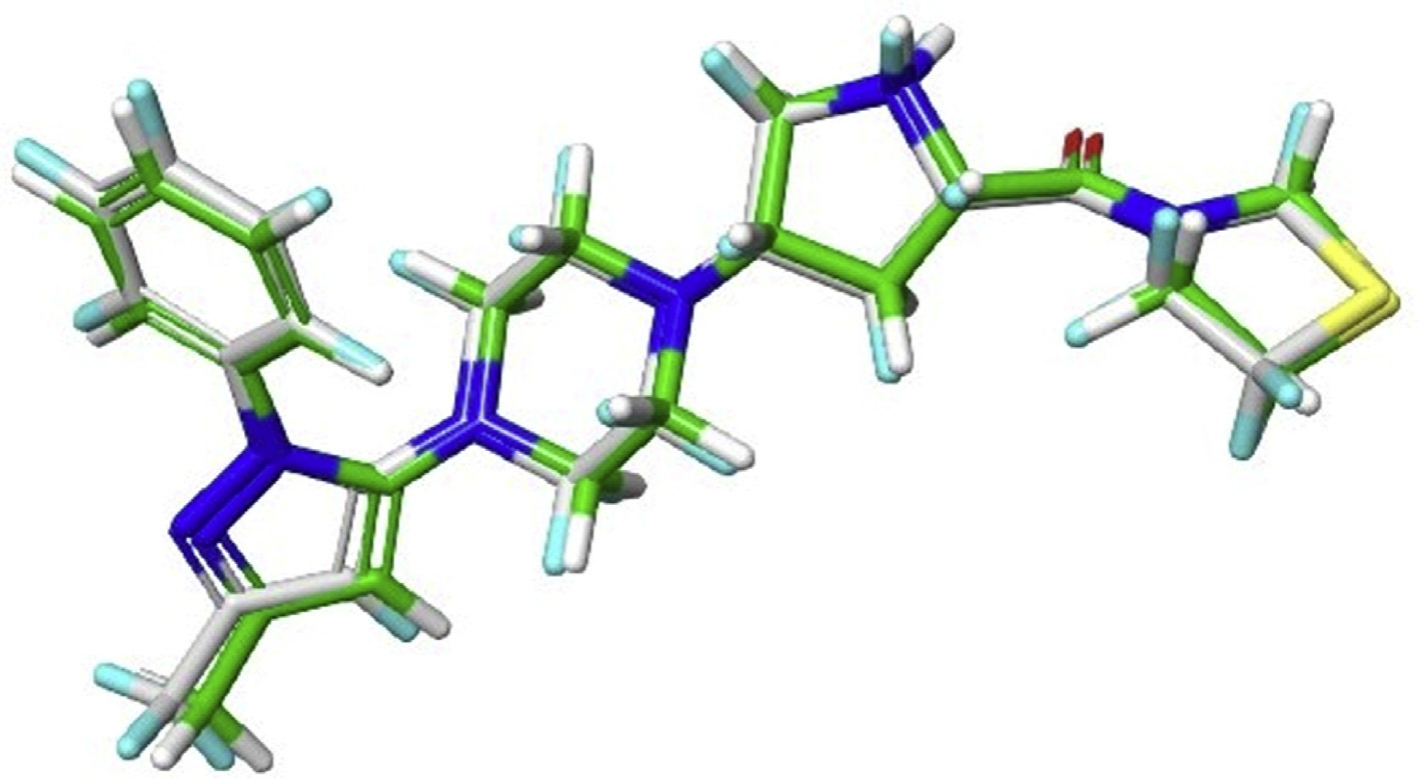
Overlay of Co–crystal pose (Green) with docked pose (white) of Teneligliptin in the active site of DPP-IV (PDB ID: 3VJK).

**Fig. 3:**
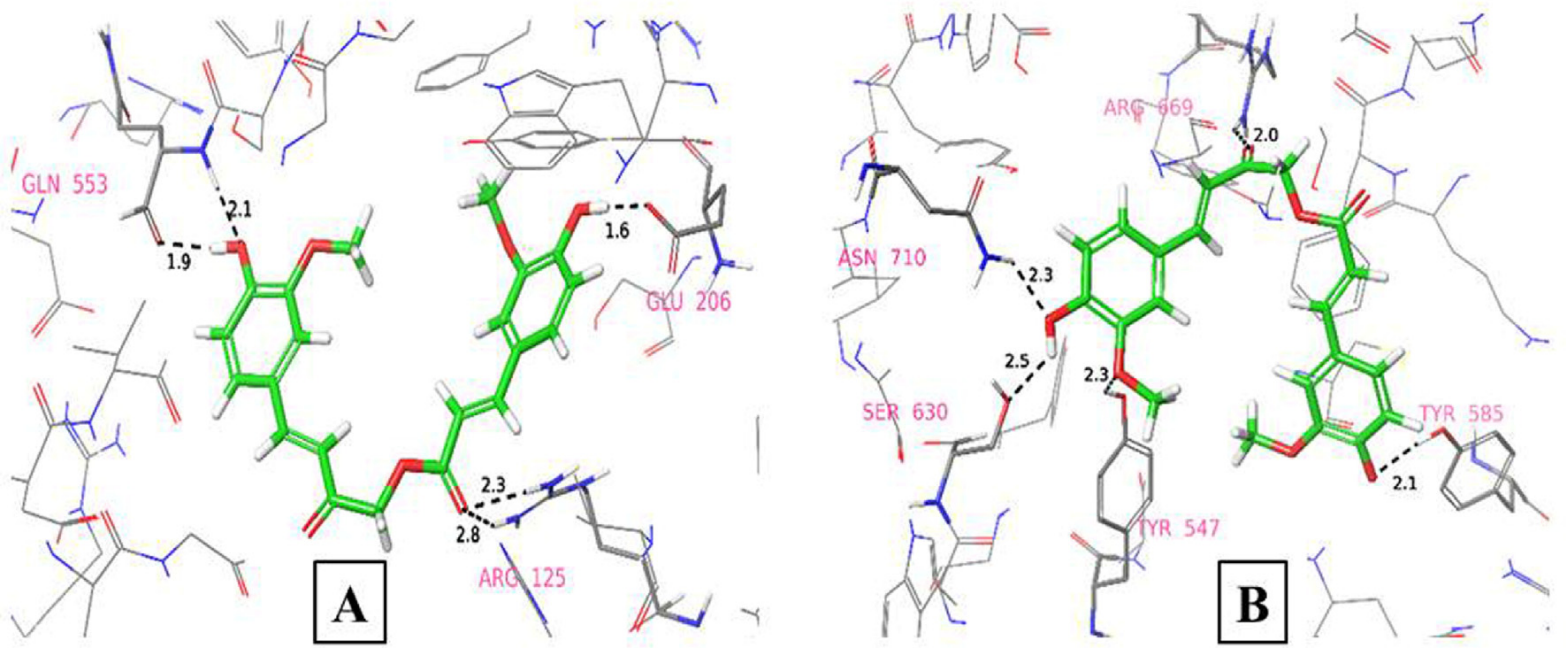
Binding poses of Ligprep generated conformations of CA in the active site of DPPIV (PDB ID: 3VJK) having glide scores of (A) with −6.28 kcal/mol and (B) with −2.36 kcal/mol.

It was clearly evident from the Ramachandran plot, that 95% and 5% of residues are in favoured and allowed regions respectively, and none of the residues were found to be in disallowed region (Fig. 1). The docking algorithm was validated by its ability to reproduce the interactions between native and low energy binding pose of co–crystal ligand. The RMSD value of 0.77 Å, that was noted during the process of validation (by overlapping native and low energy poses) indicate the reliability of glide docking method (Fig. 2). Two ligprep generated conformations have shown glide g-score of −6.284 kcal/mol and −2.369 kcal/mol with glide energy of −46.774 kcal/mol, and −37.512 kcal/mol respectively when subjected for XP docking. The conformer with high glide g-score, forms five hydrogen bonds with Glu206, Arg125, Gln553, and other conformer with low glide g-score also forms five hydrogen bonds but with Tyr547, Ser630, Asn710, Arg669, and Tyr585 (Fig. 3). The highest ranked conformer was subjected for further studies.

**Fig. 4:**
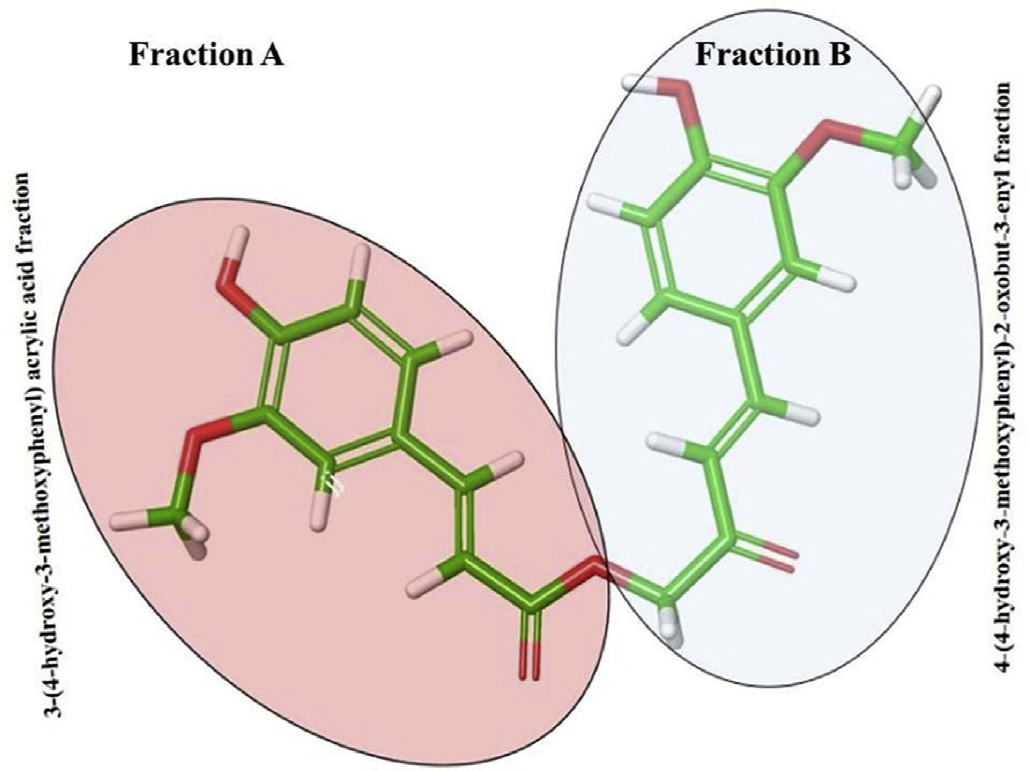
CA –Structural fractions (Fraction A and Fraction B).

The highest ranked (IFD Score: −30984.607) ligand–protein complex predicted by IFD protocol revealed that, CA forms seven hydrogen bonds with the active site residues of DPP-IV whereas, side-chain carboxylate oxygen (O=C–O^–^--- H–O, 1.7 Å) and back-bone carbonyl group (-C=O ⋯ H–O, 2.0 Å) of Glu206 stabilizes the ligand by forming two hydrogen bonds with two hydroxyl groups on each aromatic ring. Side chain hydroxyl group of Tyr662 forms two equidistant hydrogen bonds with methoxy (O–H ⋯ O–CH_3_, 2.1 Å) and hydroxyl (O–H ⋯ O–H, 2.1 Å) oxygens of 4-Hydroxy-3-methoxyphenyl ring of 4-(4-hydroxy-3-methoxyphenyl)-2-oxobut-3-enyl fraction (here after fraction A) (Fig. 4). The intermediate hydrocarbon chain was positioned by forming three hydrogen bonds, one between carbonyl oxygen of fraction A and side chain hydroxyl group (O–H⋯O=C-, 2.2 Å) of Ser552 and other carbonyl oxygen of 3-(4-hydroxy-3-methoxyphenyl) acrylic acid fraction (here after fraction B) (Fig. 4) forms two hydrogen bonds with thiol (SH⋯ O=C-, 2.7 Å) of Cys551 and hydroxyl group (O–H⋯O=C-, 1.8 Å) of Tyr 585 (Fig. S1 and S2). Free energy calculation studies by MMGB-SA showed that non-bonding interactions like Coulombic (ΔG bind_coul_: −101.73 kcal/mol), Lipophilic (ΔG bind_lipo_: −39.88 kcal/mol), and van der Waals (ΔG bind_Vdw_: −54.06 kcal/mol) energies are favouring towards overall free energy of binding with ΔG bind value of −98.72 kcal/mol.

### 3.2 Inhibition assay results

**Fig. 5:**
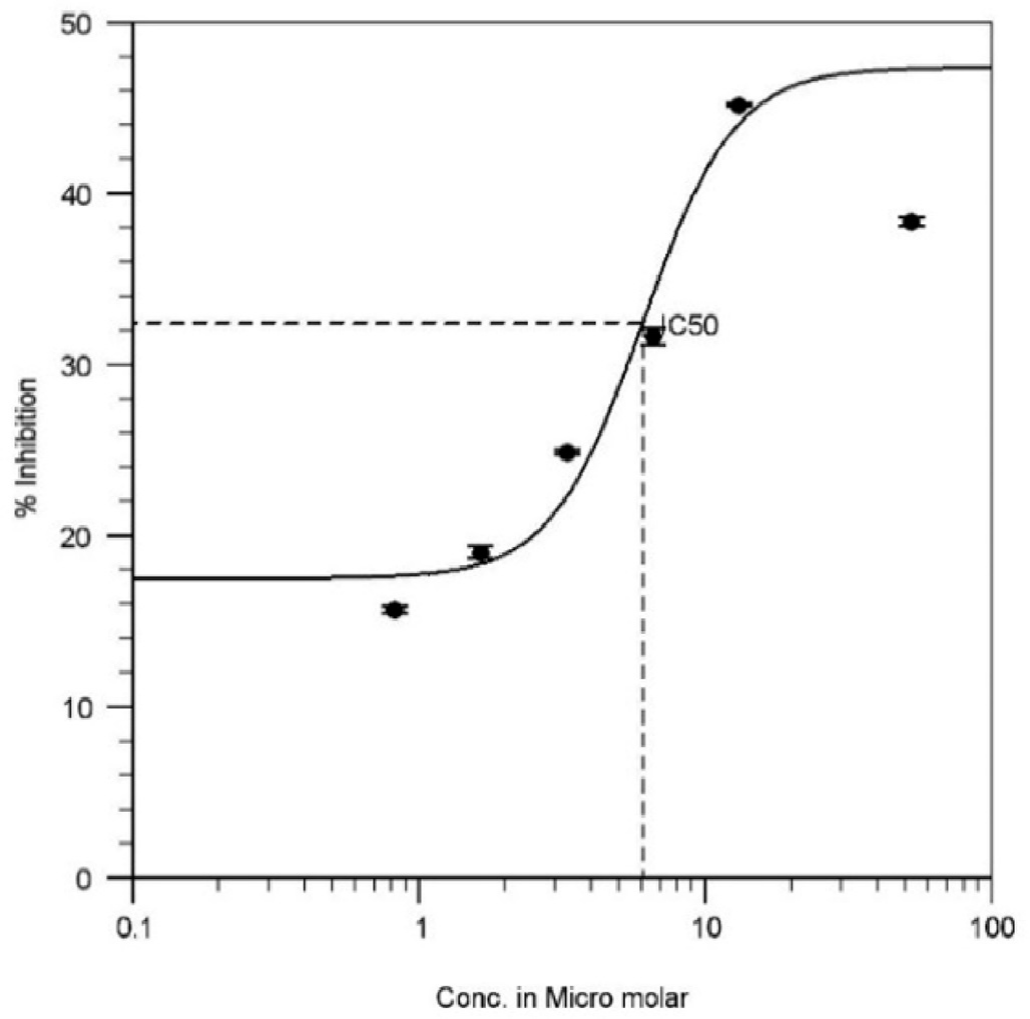
IC_50_ plot of CA.

Results were analysed through regression analysis using GraphPad Prism 6.0 and IC_50_ was found to be 6.09 ± 0.41 μM with a maximum % inhibition of 55.9 at 26.3 μM with dose-dependent inhibition (below mentioned graph – best fit; Fig. 5). This DPP-IV inhibitory activity might be due to the possible interactions shown by CA, that were revealed through *in* *silico* studies.

Standard drug sitagliptin (provided with kit) showed the % inhibition of 82.83 ± 0.08 at a final concentration of 100 μM (as per the kit). Robustness of the experimentation was measured in terms of Zʹ by using the formula that was provided by the manufacturer. In the current investigation Zʹ was found to be 0.84 (robust assay has >0.5).

$$Z^{\prime }=1-\frac{3\sigma_{\text{c}+}+3\sigma_{\text{c}-}}{\left|\mu_{\text{c}+}-\mu_{\text{c}-}\right|}$$

σ: Standard Deviation

μ: Mean

c+: Positive control

c-: Negative control.

### 3.3 Molecular dynamic simulations results

**Fig. 6:**
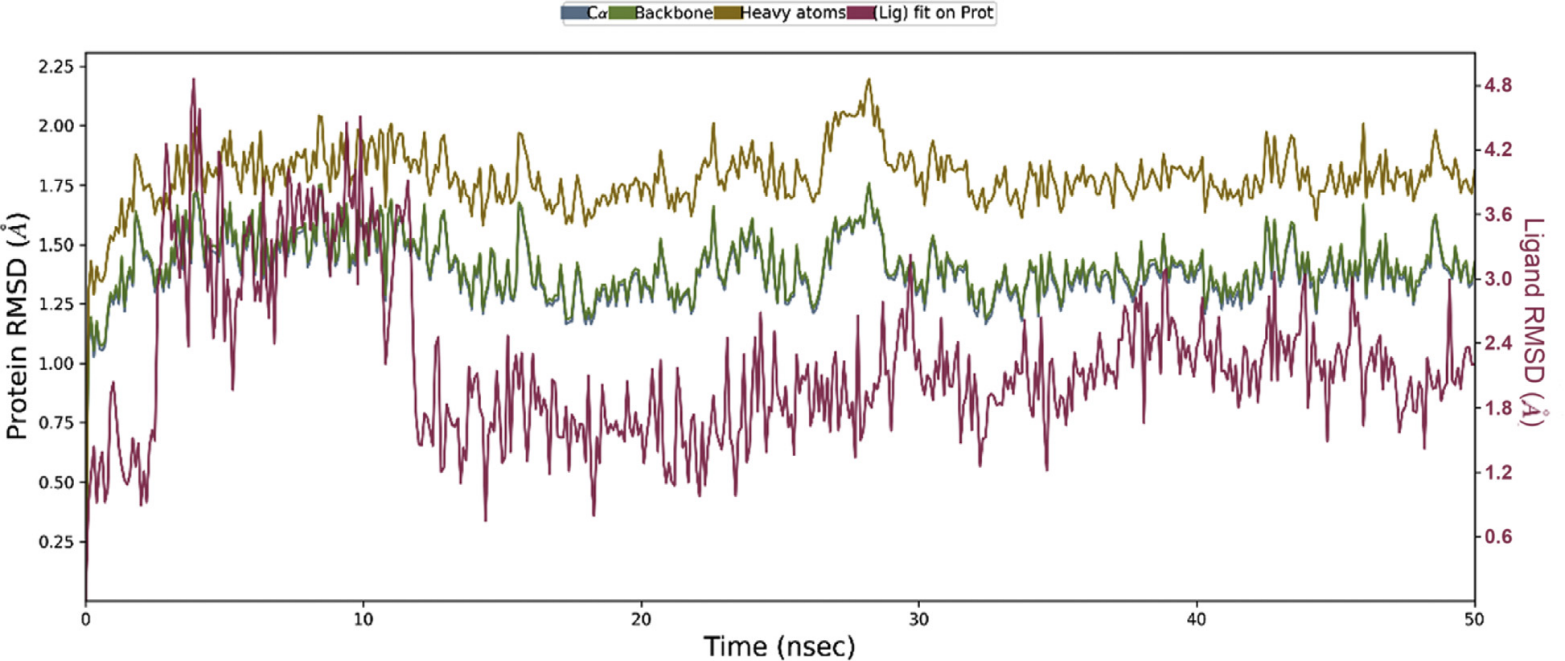
RMSD plot for the Protein Cα, Backbone, heavy atoms and co–crystal ligand.

The RMSD plot of ligand free apoenzyme (Fig. S3) has clearly showed the acceptable range of elevation in the Cα-RMSD value for a period of 50 ns as 1.3–2.7 Å. On the other hand, simulation results (for cocrystal) revealed the fluctuations in RMSD of Cα (1.03–1.44 Å) and backbone (1.21–1.58 Å) for a period of first 10 ns (i.e., 1–10ns), after which the RMSD got stabillise (Cα 1.31–1.75 Å and backbone 1.35–1.76 Å) as the simulation proceeds till 50 ns. (Fig. 6).

Through molecular dynamics simulation, the binding phenomenon of CA in relation to the stability of the protein was studied for a period of 50 ns. Based on the conformational changes in the binding mode of the ligand, the whole trajectory was divided into two-time lines: 1) Stability of ligand in the active site up-to 33 ns - Initial phase 2) High conformational flexibility of ligand from 34 ns to end of simulation- Final phase.

#### 3.3.1 Initial phase

The conformational flexibility of ligand was studied at 5 ns interval of MD trajectory (Supplementary data). In the initial binding mode (Fig S4), the side chain and backbone oxygens of Glu206 formed two hydrogen bonds with hydroxyl groups of fraction A (1.8 Å) and B (1.9 Å) firmly holding the ligand to appear like a bow shape. The 4-hydroxy-3-methoxy phenyl group of fraction A was further positioned by two hydrogen bonds (2.1 and 2.0 Å) with side chain hydroxyl group of Tyr 662 along with the formation of π-cation interaction with Arg125. Two carbonyl oxygens of the (ligand) hydrocarbon chain formed the hydrogen bonds with Tyr547 and Ser552 at a distance of 2.2 and 1.9 Å respectively.

From Fig S5 & S6, it was evident that, 4-hydroxy-3-methoxyphenyl ring of fraction B seems to be fluctuating away from the initially established contact i.e., backbone carbonyl oxygen of Glu206 and observed to form near distant hydrogen bonds with the side chain of Arg358 at 5 ns (1.9 Å) and 10 ns (2.3 Å). This fluctuation resulted in an increase in distance between hydrocarbon chain and residues Tyr547 and Ser552. The side chain carboxyl group of Glu206 formed two hydrogen bonds with hydroxyl group of fraction A. Although the fluctuations created the alterations in the interactions of fraction B, 4-hydroxy-3-methoxy phenyl group of fraction A continued to maintain its hydrogen bonds with side chain hydroxyl group of Tyr662. Further, the ligand was positioned in the active site by another interaction at 10 ns, between Val207 and hydroxyl group of fraction B (2.2 Å) (Fig S6).

From 15 to 30 ns (Figure S7, S8, S9 and S10), despite persistent fluctuations [which may be due to methylene (–CH_2_–) bond rotation], hydrogen bonds formed by side chain hydroxyl group of Tyr662, backbone carboxyl group of Glu206 and side chain amino group of Arg358 with 4-hydroxy-3-methoxy phenyl rings of fractions A and B might be responsible in positioning the ligand in the active site of an enzyme to maintain considerable stability. Apart from these, hydrophobic interactions between Tyr666 and phenyl ring of fraction A also contributed for the same.

From 31 to 33 ns (Figures S11, S12 and S13), the 4-hydroxy-3-methoxy phenyl rings of fractions A and B were gradually displaced away from the interacting residues viz., Glu 206, Tyr662, and Arg358 but maintained hydrogen bonds with Tyr547 and Ser552 with little fluctuations. However, Tyr666 continued to maintain hydrophobic contact (π-π) with phenyl ring of fraction A.

#### 3.3.2 Final phase (34–50 ns)

From 34 to 50 ns, the high conformational flexibility of ligand (Figures S14, S15, S16, S17 and S18) caused the rotation of phenyl rings and resulted in the formation of new hydrogen bonds with residues Ala654, Val656, and Asn710 of S_1_ subsite followed by hydrophobic contact with Phe357.

**Fig. 7:**
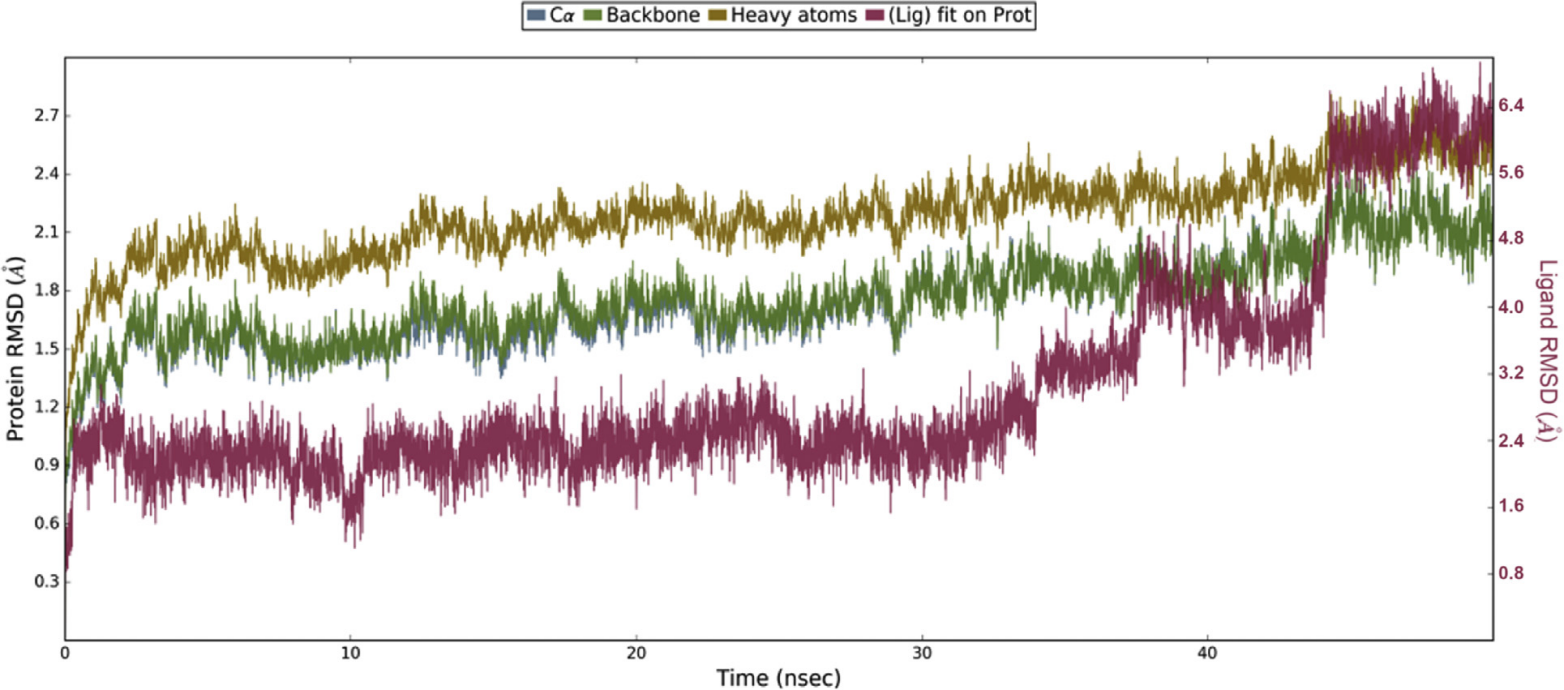
RMSD plot for Protein Cα, Backbone, Heavy atoms and Ligand.

Initially, there was an increase in protein RMSD during first 2 ns simulation, which might be due to equilibration of protein-ligand complex with the solvent system. From 2 ns to 50 ns (Fig. 7), the RMSD of protein Cα and backbone were observed in the range of 1.68–2.5 Å and 1.53–2.3 Å respectively. In the RMSD of protein, fluctuations were noticed in Cα and backbone up to 29 ns after which gradually increased to 1.99 Å and 1.94 Å respectively at 33 ns, and then reached to 2.5 Å and 2.3 Å respectively at 44 ns. Finally, there was downshift in RMSD values of 2.18 and 2.01 Å.

In ligand RMSD, high fluctuation was observed in the range of 2.18–6.58 Å. Initially there was an increase in ligand RMSD having 2.48 at 0.36 ns and then maintained in the range of 2.18–2.88 Å from 10 to 34 ns. After 34 ns, the ligand RMSD was airlifted from 3.0 Å and noticed maximum value of 6.57 Å at 48 ns. The root mean square fluctuations (RMSF) for the amino acid residues of protein Cα, backbone and heavy atoms were observed in the range of 0.52–3.51 Å, 0.53–3.47 Å, and 0.59–4.06 Å respectively (Fig S19). High root mean square fluctuations were observed for the residues, Ser245 (Cα- 3.51 Å, backbone- 3.47 Å), Gln247 (Cα- 3.40 Å, backbone- 3.31 Å), Val279 (Cα- 2.85 Å, backbone- 2.85 Å) and Glu677 (Cα- 2.86 Å, backbone- 2.86 Å) and low root mean square fluctuations were observed for the residue, Leu543 (Cα- 0.56 Å, backbone- 0.56 Å).

**Fig. 8:**
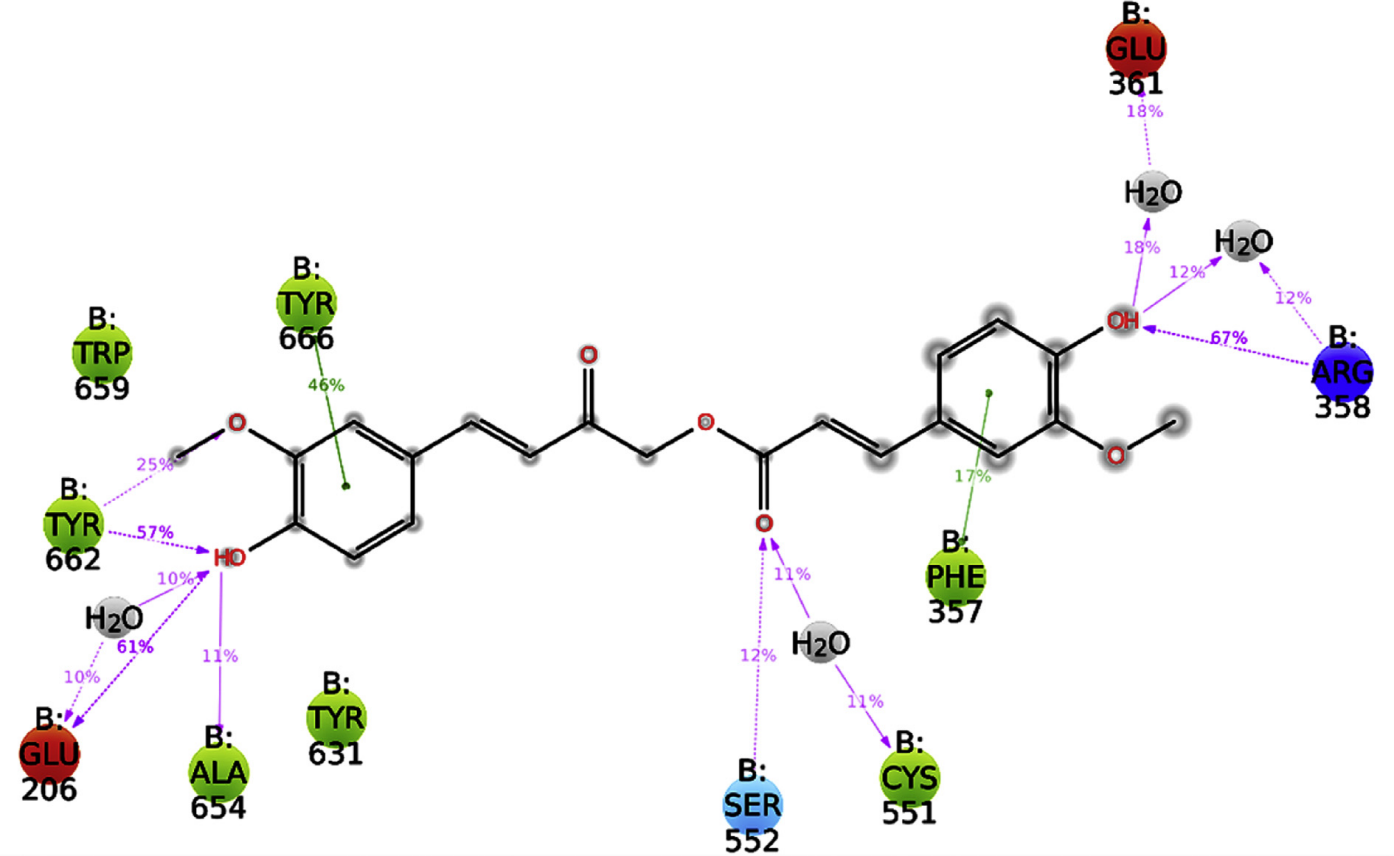
2D Binding interaction diagram of CA throughout MD simulation.

The trajectory analysis of MD simulation (Fig. 8) conveyed that CA interacted with various residues of subsites present in the active site of the enzyme. It was noted that Glu206 forms two hydrogen bonds with hydroxyl group of fraction A viz., one moderately strong hydrogen bond between hydroxyl group and side chain carboxylic oxygen of Glu206 (HO---^–^OOC) (61% of MD trajectory) and another weak bond through a water bridge (20% of MD trajectory). The backbone hydroxyl group of Tyr 662 forms two hydrogen bonds, with methoxy oxygen (H_3_CO---HO-, 25% of MD trajectory) and hydroxyl oxygen (HO⋯HO-, 57% of MD trajectory) of fraction A in a bidentate manner. Another key residue, Arg358 was also noticed to form two hydrogen bonds with hydroxyl group of fraction B, one moderately strong hydrogen bond with hydroxyl group (HO⋯NH-, 67% of MD trajectory) and a weak bonding through a water bridge (24% of MD trajectory). The phenyl rings of fraction A and fraction B were positioned in the active site through hydrophobic interactions with Tyr666 (46% of MD trajectory) and Phe357 (17% of MD trajectory) respectively. In addition, Ser552 (12% of MD trajectory), Ala654 (11% of MD trajectory), Cys551 (water bridging, 22% of MD trajectory), and Glu361 (water bridging, 36% of MD trajectory) showed weak interactions with CA.

As represented in Fig S20, radius of gyration (rGyr), Molecular surface area (MolSA), Solvent Accessible Surface Area (SASA) and Polar Surface Area (PSA) of the ligand were observed in the range of 4.87–6.38 Å, 372.66–385.56 Å^2^, 126.18–273.86 Å^2^ and 180.98–204.9 Å^2^ respectively.

## 4 Discussion

The enzyme DPP-IV belongs to the superfamily of prolyl oligopeptidase and differs from other oligopeptidases in having two glutamate residues (Glu205 and Glu206) in the catalytic pocket of active site that have their own niche role in exhibiting enzymatic activity [28]. Glucagon like peptide-1(GLP-1) got its significance after the discovery of stimulator action towards insulin release in the late 1980s. It gets inactive by DPP-IV within 2 min from its GLP-1(7–36) form (active) to GLP-1(9–36) form (inactive), that lacks agonist action towards its own receptor. DPP-IV inhibitors enhance the homeostatic environment by elevating the life span of GLP-1 [29,30]. The well tolerability of DPP-IV inhibitors makes them more vulnerable. Various clinical trials had emphasized the lack of considerable difference in the incidence of adverse effects between comparator (placebo) and inhibitor groupings [31]. Moreover, DPP-IV inhibition may not affect its role in the immune system as the enzyme appears to be involved in the two activities independently [32,33]. Several molecules have been proved for DPP-IV inhibitory action so far, with various potencies through different modes of binding. Initially designed molecules were called as peptidomimetics due to the presence of proline mimicking dipeptide at P1 position. However, due to the lack of considerable chemical stability, they lost their significance. By considering this issue, several molecules had come up that can interact with various subsites as well as extended sites of the active site [[34], [35], [36]].

For the current study, constituents of *C. longa* were selected. From ancient times, *C*. *longa* has its own niche in promoting health. Due to advances in scientific field, many therapeutic actions were proved and many evidences attributed the curcumin to most of the positive actions of *C*. *longa.*

The current research work is a continuation of our repurposing strategy to our earlier research works that involved in targeting DPP-IV inhibitors for Alzheimer's disease [14,37,38]. CA stands in place after curcumin in *in* *silico* analysis for the selected receptor. In the current investigation, we have used CA for which except Aβ disaggregating property (*in vitro*) no other anti-Alzheimer's property has been described.

The active site of DPP-IV was constructed into different subsites based on substrate and inhibitor binding with respective amino acid residues, they are S_2_ extensive subsite (Val207, Ser209, Phe357, Arg358), S_2_ subsite (Arg 125, Phe357, Arg358, Glu205, Glu206, Arg669), S_1_ subsite (Ser630, Val656, Trp659, Tyr662, Tyr666, Val711, Asn710), S′_1_ subsite (Tyr547, Ser630, Phe357, Pro550, Tyr631, Tyr666), and S′_2_ subsite (Tyr547, Trp629, Ser 630, His740) [[39], [40], [41]]. Non-bonded interacting residues with respect to energy and distance involved for binding of CA were mentioned in Table 1. Binding mode of CA in the active site of DPP-IV at subsite level was represented by Fig S21 (rendered by PyMOL-v 1.3) [42].

**Table 1:** Non-bonded interaction energies involved in binding of CA-DPP-IV (IFD complex).

S.No	Residue	Vdw (Kcal/mol)	Coul (Kcal/mol)	Dist. (Å)	Eint (Kcal/mol)
S_2_ extensive subsite					
1	Val207	−0.39	0.15	4.825	−0.242
2	Ser209	−1.86	−0.99	2.763	−2.864
3	Phe357	−5.31	−0.63	2.508	−5.945
4	Arg358	−2.48	−5.08	2.044	−8.475
S_2_ subsite					
1	Arg125	−0.98	2.5	2.408	1.526
2	Glu205	−1.83	−1.78	2.758	−3.623
3	Glu206	−2.38	1.2	2.92	−1.176
4	Arg669	−0.3	0.03	4.351	−0.275
S_1_ subsite					
1	Ser630	−1.54	0.22	3.013	−1.328
2	Val656	−0.71	0.001	2.378	−0.712
3	Trp659	−0.64	−0.04	2.452	−0.688
4	Tyr662	−2.67	−0.04	2.796	−2.719
5	Tyr666	−3.33	−0.13	2.753	−3.467
6	Asn710	−1.59	−2.52	2.047	−5.12
7	Val711	−0.83	−0.03	2.338	−0.867
S′_1_ subsite					
1	Tyr547	−0.89	−0.29	2.631	−1.188
2	Tyr631	−1.6	−0.46	3.069	−2.069
3	Pro550	−0.25	−0.03	4.716	−0.293
S′_2_ subsite					
1	His740	−0.91	−0.03	3.534	−0.948
2	Trp629	−0.12	0.11	6.988	−0.006

Vdw: Van der Waals interaction between the residue and the ligand.

Coul: Electrostatic interaction between the residue and the ligand.

Dist.: Minimum distance between the residue and the ligand.

Eint: Sum of the Coulomb and van der Waals energies (non-bonded interaction energy).

The qualitative and quantitative investigations like comparative binding analysis of DPP-IV inhibitors with their inhibitory activities based on X-ray crystal poses [41], binding kinetics, and thermodynamics approach towards structural relationship of DPP-IV inhibitors using surface plasmon resonance (SPR) and isothermal titration calorimetry (ITC) [43], *in* *silico* insights in molecular design of DPP-IV inhibitors [44] and quantitative estimation of interaction energies using fragment molecular orbital (FMO), and quantum-mechanical (QM) methods [45] disclosed that hydrophobic interactions play a decisive role in DPP-IV inhibitory activity along with increase in number of interactions into their respective subsites with increase in molecular surface area of inhibitors. In connection to the above conclusions, we performed a preliminary *in* *silico* docking and free energy minimization studies to analyze binding interactions of CA with DPP-IV at subsite level. Having molecular surface area of 378.778 Å^2^, the 4-hydroxy and 3-methoxy groups of fraction A occupied S_1_ subsite via formation of two hydrogen bonds with Tyr662. However, van der Waals dispersion (hydrophobic) [45] interactions were also observed with Tyr662 (−2.673 kcal/mol) and Tyr666 (−3.334 kcal/mol) (Table 1). The two hydroxyl groups of CA (from fraction 1 and fraction 2) occupied S_2_ subsite forming two significant hydrogen bonds with side chain and backbone of Glu206. The carbonyl oxygens of both fractions formed three hydrogen bonds with Cys551, Ser552 and Tyr585.

**Fig. 9:**
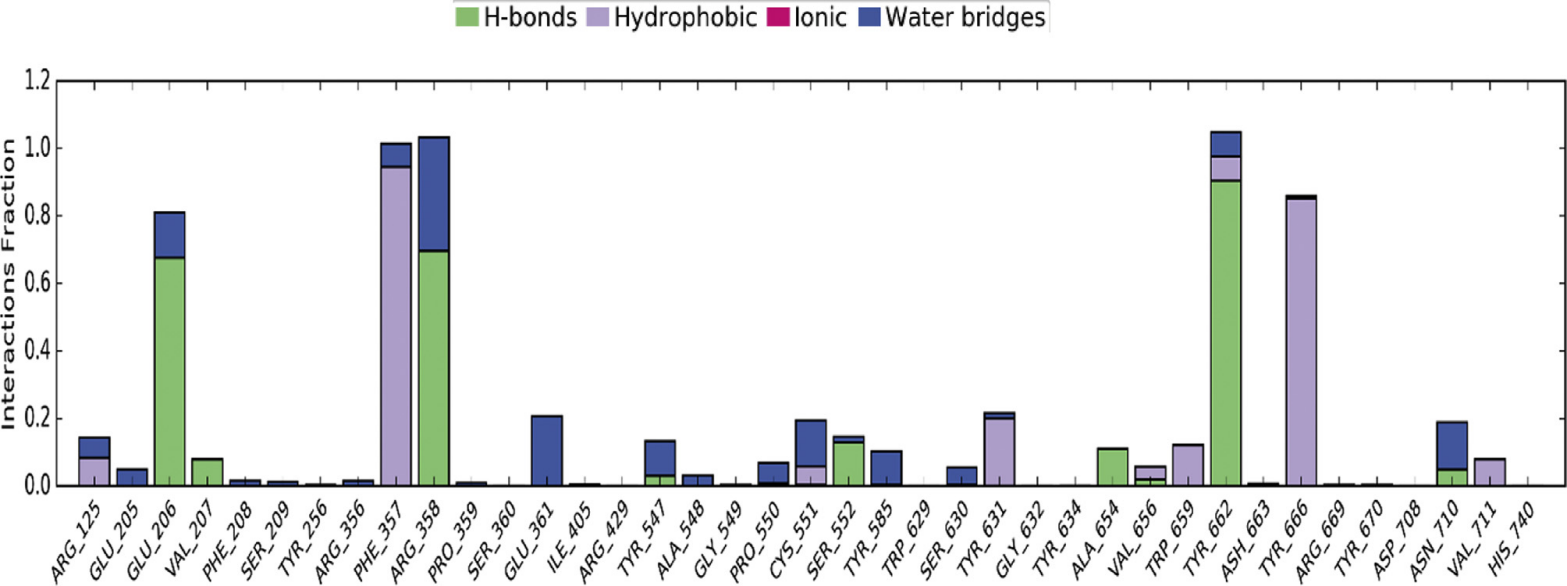
Interaction fraction of CA with active site residues of DPP-IV.

Apart from these findings, CA also showed non-bonded interactions like van der Waals dispersion (hydrophobic) with Phe357 (−5.313 kcal/mol) and Coulombic interactions with Arg358 (−5.089 kcal/mol) (Table 1), through π-π and π-cation interactions respectively, due to phenyl ring occupation into S_2_ extensive subsite. It was also reaffirmed by the trajectory analysis of 50 ns MD simulation (Fig. 9) (revealed) that CA was stabilized in the active site by forming various bonded (hydrogen bond) and non-bonded (Vander waal Dispersion (hydrophobic), Coulombic) interactions with key residues like Glu206, Phe357, Arg358, Tyr662, and Tyr666 as shown in Fig. 9. From the data, it was clear that hydrogen bonding interactions with Glu206, Arg358, and Tyr662 followed by hydrophobic contacts with Phe357 (not represented in images) and Tyr666 played a key role in stabilising the ligand in the active site (up to 34 ns). During the final phase of simulation, from 34 to 50 ns, the ligand was completely displaced form the active site by forming new bonds with Ala654, Val656 and Asn710 followed by hydrophobic contact with Phe357. However, this high conformational flexibility of ligand throughout the simulation had moderate influence on the RMSD of protein Cα and backbone showing difference in fluctuation of 0.82 Å and 0.77 Å respectively.

**Fig. 10:**
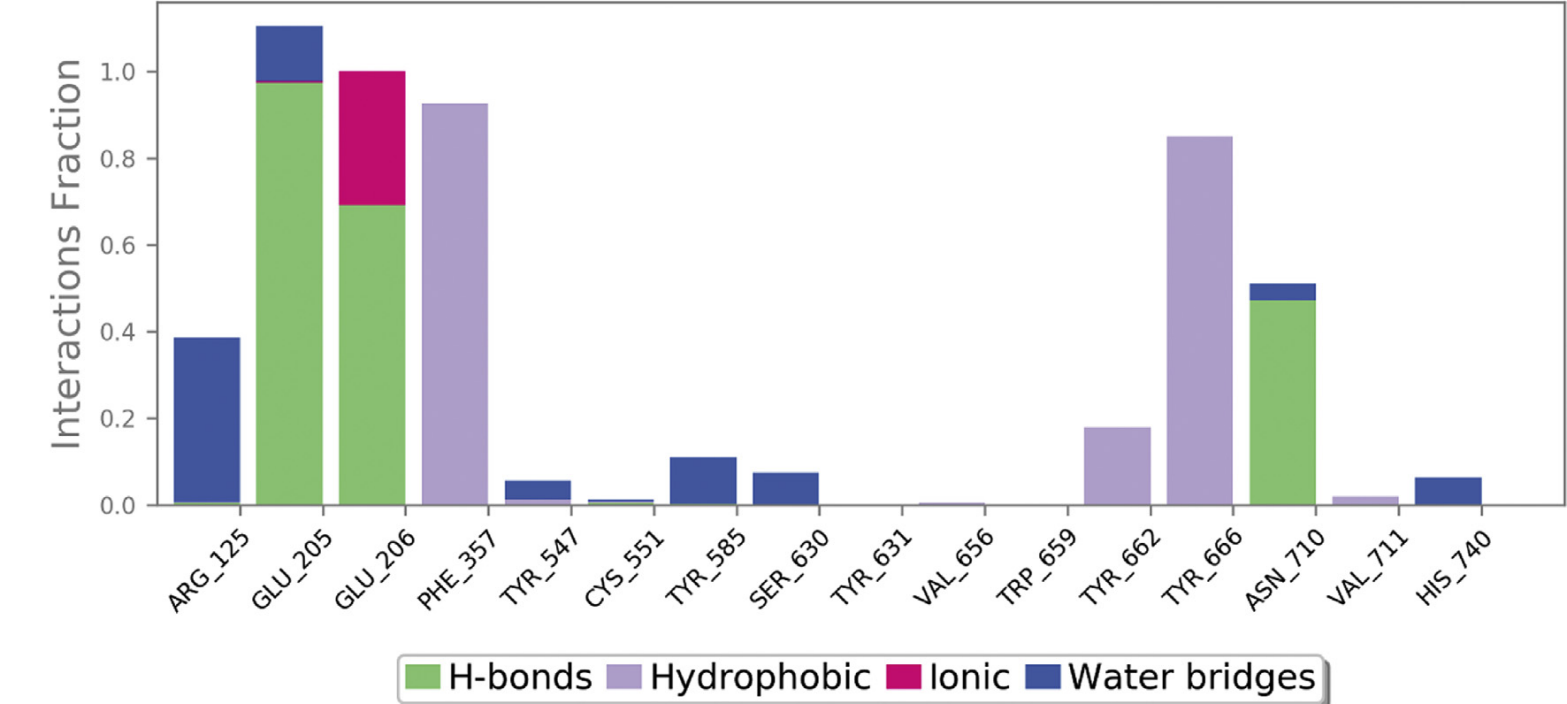
Interaction plot of teneligliptin with different catalytic pocket residues of DPP-IV (PDB ID: 3VJK) throughout the 50 ns simulation trajectory.

**Fig. 11:**
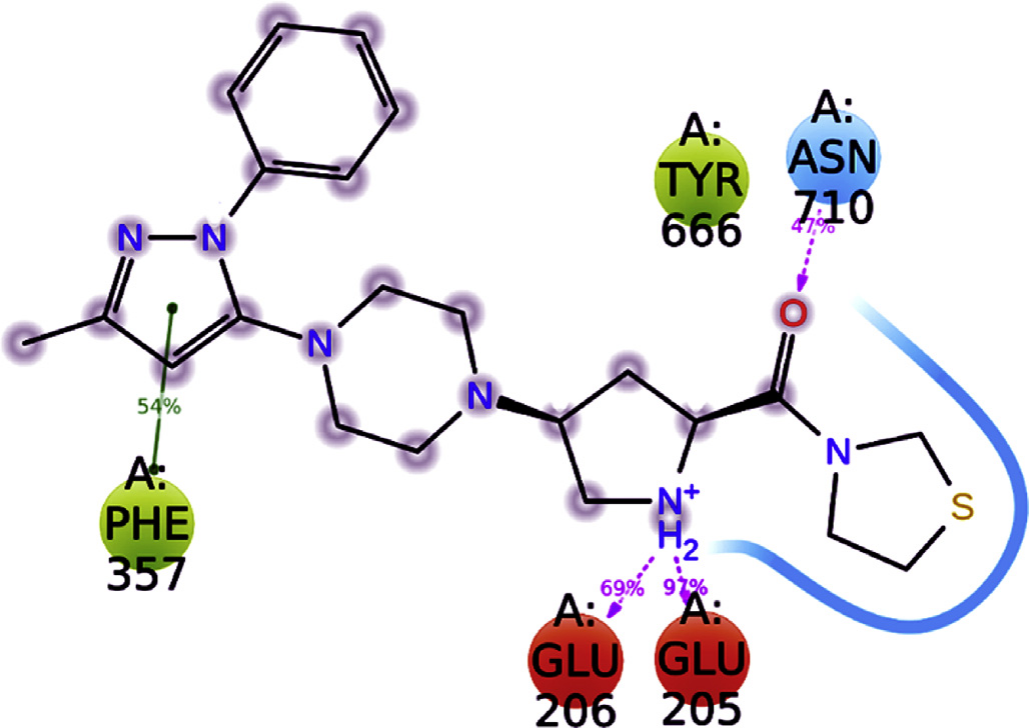
2D-interaction diagram of teneligliptin in the catalytic pocket of DPP-IV (PDB ID: 3VJK) throughout the 50 ns simulation trajectory.

In case of co–crystal (teneligliptin) also, both bonded and non-bonded interactions played a key role in stabilising the molecule. The ligand bound amino acids displayed low RMSF (Fig S22) of 0.43–0.66 Å indicating low fluctuations of residues caused by tight binding with teneligliptin. From Fig. 10, it was clear that Glu205, Glu206, and Asn710 formed multiple contacts like hydrogen bonding, ionic interactions, and water bridges with teneligliptin throughout 50 ns simulation trajectory. However, Phe357, Tyr662 and Trp666 formed hydrophobic contacts. From the 2D interaction diagram (Fig. 11) of 50 ns trajectory, it was observed that carbonyl oxygen of tenelegliptin formed one low frequency hydrogen bond (47% of trajectory) with Asn710. On the other hand, two hydrogens of protonated amino group in pyrrolidine ring formed moderate and high frequency hydrogen bonds at 69 and 97% of trajectory respectively with Glu206 and Glu205. The pyrazole ring is stabilized in its position by forming π- π stacked bonding with Phe357 at 54% of total trajectory.

In most research studies, related to herbal DPP-IV inhibition activity, extracts were tested for the activity and even if the individual components subjected for the same, progressing them for *in silico* studies is rare, as the usage of *in silico* technology in the process of drug discovery is emerging now. This might be one of the main reasons for the lack of comparison data among natural DPP-IV inhibitors. Divya et al., reviewed and compiled the data related various natural extracts and compounds that were proved to have DPP-IV inhibition activity through *in* *vitro* studies [46]. As proposed by Arulmozhiraja et al. interaction with Glu205, Glu206 besides Tyr547, Trp629, Tyr666, and Phe357 is an important criterion for the DPP-IV inhibitory action of existing market products such as sitagliptin, alogliptin, linagliptin, and teneligliptin, substantiates the current research study [47]. Binding of curcumin in the S1 subsite (besides interactions with Glu205, Glu206) attributed for the DPP-IV inhibitory action was proved by Huang et al.*,* further supports the current research work [48]. *In silico* studies on stellasterol isolated from *Ganoderma australe* highlighted the importance of hydrophobic interactions with Tyr631, Tyr662, Trp659, Tyr666, and Val711 for DPP-IV inhibition which further strengthen the current investigations [49]. Other natural compounds like malonylgenistein, Caffeic acid – 3-glucoside and Calenduloside E were proved to have interactions similar to that of CA [50].

Although DPP-IV inhibitors can be screened by direct enzymatic, *ex vivo and in vivo* methods, direct enzymatic method is an ideal and appropriate method in the initial stages of drug discovery and development process (as many number of compounds can be screened). Due to the limited number of DPP-IV inhibitors in the market, Lin et al., emphasized importance of considering the natural compounds in the drug discovery process and the same can be hastened by implementing *in silico* and enzyme inhibition methods [51]. In another research, Idowu et al.*,* identified the hypoglycemic activity of *Brachylaena elliptica* is independent of insulin which might be due to its DPP-IV inhibition activity that was proved through *in* *vitro* study [52]. The research work by Poonam et al., revealed the DPP-IV inhibitory potential of garlic extract through *in vitro* methodologies [50]. In general, the quality of enzymatic assays or *in vitro* assays is measured by statistic parameter called Z'(Z prime) which uses mean and standard deviation of both positive and negative control. Z' is not only a measure of way of doing but also correctness of instrument. Hence, in short we can consider the Z' as statistic measure of overall experimentation. In general, a good Z' value lies between 0.5 and 1. Since the current experiment has got the value of about 0.8, which indicates the quality of overall experimentation. As mentioned earlier, our research mainly focuses on repurposing strategy through DPP-IV inhibitors to target AD, by considering several evidences as facts and support. DPP-IV inhibitors enhance the levels of GLP-1 that can cross Blood brain barrier (BBB) and executes its actions through GLP1R (GLP-1 receptor), present over pyramidal neurons of the hippocampus and purkinje cells of cerebellum [53]. Out of many secondary pathways activated by GLP-1, PKC (Protein kinase C) mediated actions play prominent role in neuroprotection. Various important and considerable functions execute by PKC isoforms are modulation of synaptic transmission, promoting neuronal plasticity, neuronal metabolism etc., [54]. GLP-1 was proved for its protective role against LPS insult in astrocytes [55]. Moreover, GLP-1 mimetics ameliorated detrimental effects shown by β-amyloid on synapses [56]. By using various AD mouse models, capability of incretin analogues in promoting neurogenesis during pathological condition was proved scientifically [57,58]. The present drug CA excretes via non-renal as glucuronide metabolite when administered through oral or intravenous route [59].

## 5 Conclusion

Binding mode analysis and results of enzyme inhibition emphasised the consideration of CA for its role as a DPP-IV inhibitor. As role of GLP-1 has extended beyond glycemic actions, DPP-IV inhibitors can be expected to have crucial role in various pathological features. In our previous studies, we have emphasized the importance of these inhibitors in the field of neurodegenerative disorders.

In this current investigation, *in* *silico* methods like molecular docking, binding free energy calculation, induced fit docking and molecular dynamic simulation studies were performed to analyze binding mode of CA with DPP-IV. Having a structurally diverse scaffold from the marketed inhibitors, our studies showed possible interactions of CA with substrate specific recognition site residues like Glu206 (S_2_ subsite) and Tyr662 (S_1_ subsite) of “amino hot-spot” and also with Phe357 and Arg358 of S_2_ extensive subsite, conclusively conveying that CA has the potential to act like class-3 inhibitor according to Nabeno et al. As both *in silico* and enzyme inhibition studies clearly showed encouraging results for the proposed action, further confirmation through *in vivo* studies is essential. Furthermore, the rigidification of the CA in the active site to enhance the time of contact with inhibitory interactions with the protein and steps to enhance the bioavailability was directed for future studies to make it clinically viable.

## Source(s) of funding

The work was financially supported by Ministry of AYUSH-A (EMR scheme), Government of India, New Delhi. (Sanction Order: Z.28015/89/2014-HPC (EMR) AYUSH – A).

## Conflict of interest

None.

## Author contributions

**Nehru sai suresh**: Study execution, Literature and experimental data collection, Data Interpretation, Manuscript writing.

**Srikanth Jupudi**: *In silico* study and statistical analysis.

**Venkata Ramesh Yasam**: Data analysis and Interpretation.

**Duraiswamy Basawan**: Conceptualisation, Supervision, Fund recipient, Manuscript review.
