# Supplementary material for: Dipeptidyl peptidase-IV inhibitory action of Calebin A: An in silico and in vitro analysis
Source: J Ayurveda Integr Med. 2021 Oct 29;12(4):663–72. doi: 10.1016/j.jaim.2021.08.008 (PMC8642699; doi:10.1016/j.jaim.2021.08.008)
Supplement: Multimedia component 1 [file mmc1.docx]

**Dipeptidylpeptidase-IV inhibitory action of Calebin A: An *In Silico* and *in vitro* analysis**


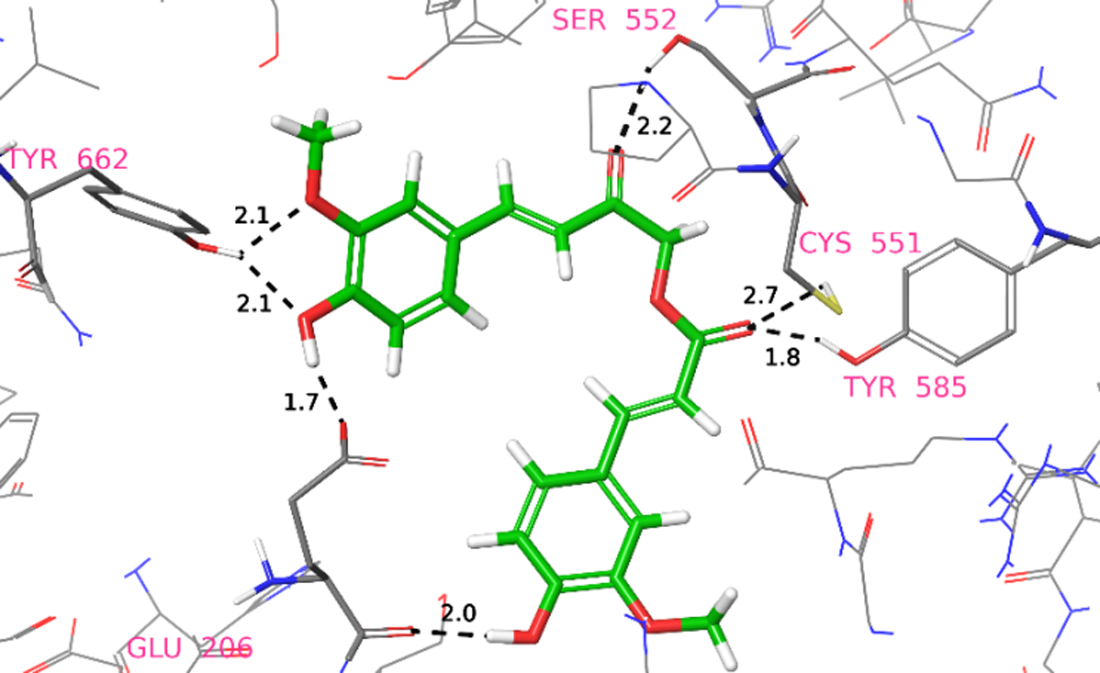


**Fig S1** IFD pose of Calebin A with binding residues in the active site of DPP-IV


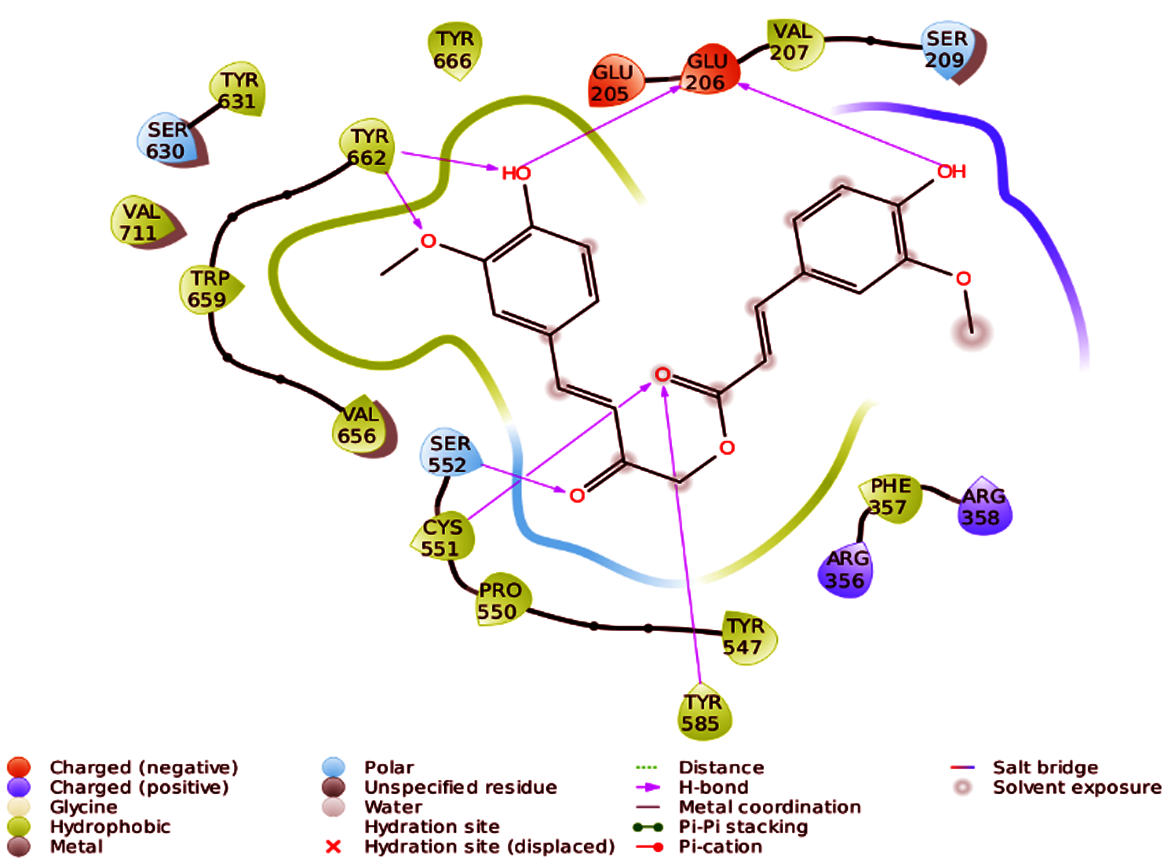


**Fig S2** 2D diagram showing Hydrophobic residues (green colour) involved in binding of Calebin A.


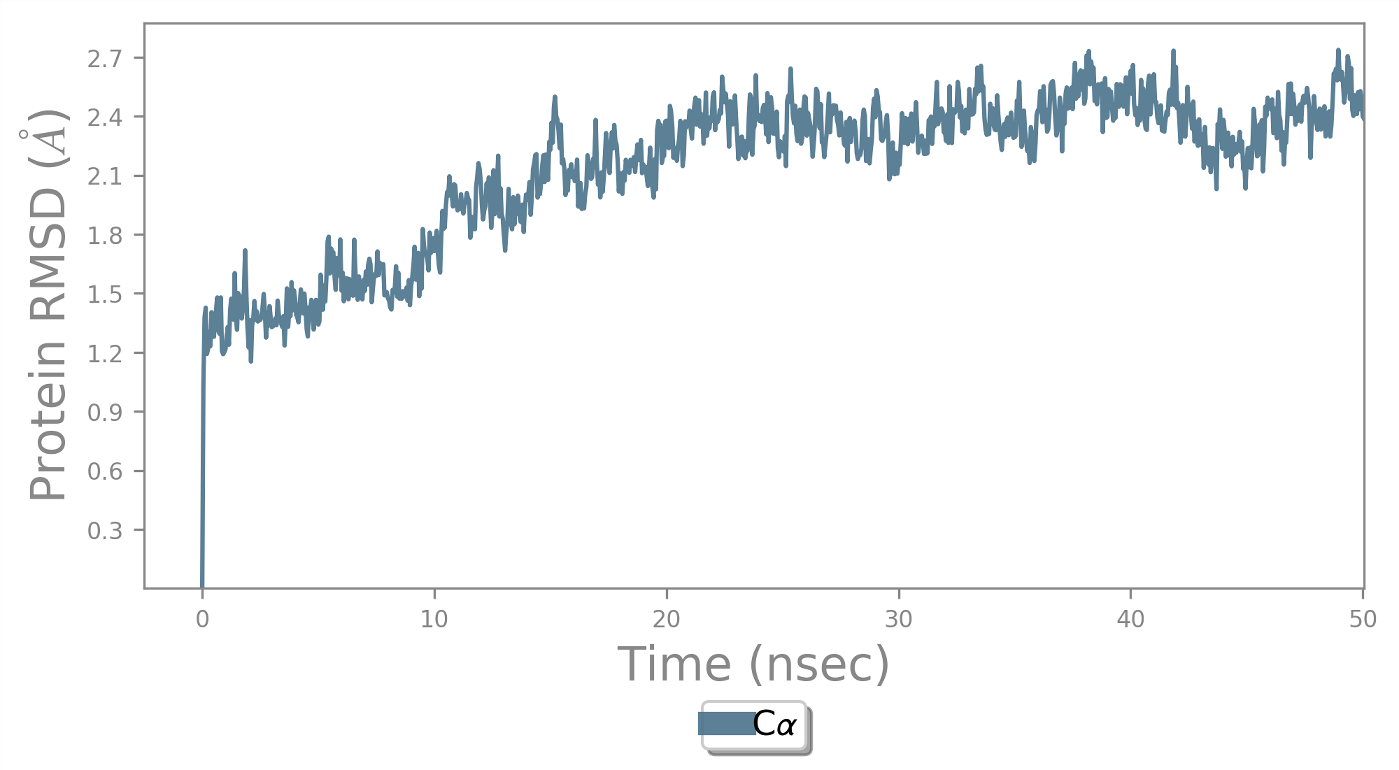


**Fig S3** RMSD of protein Cα for apoenzyme


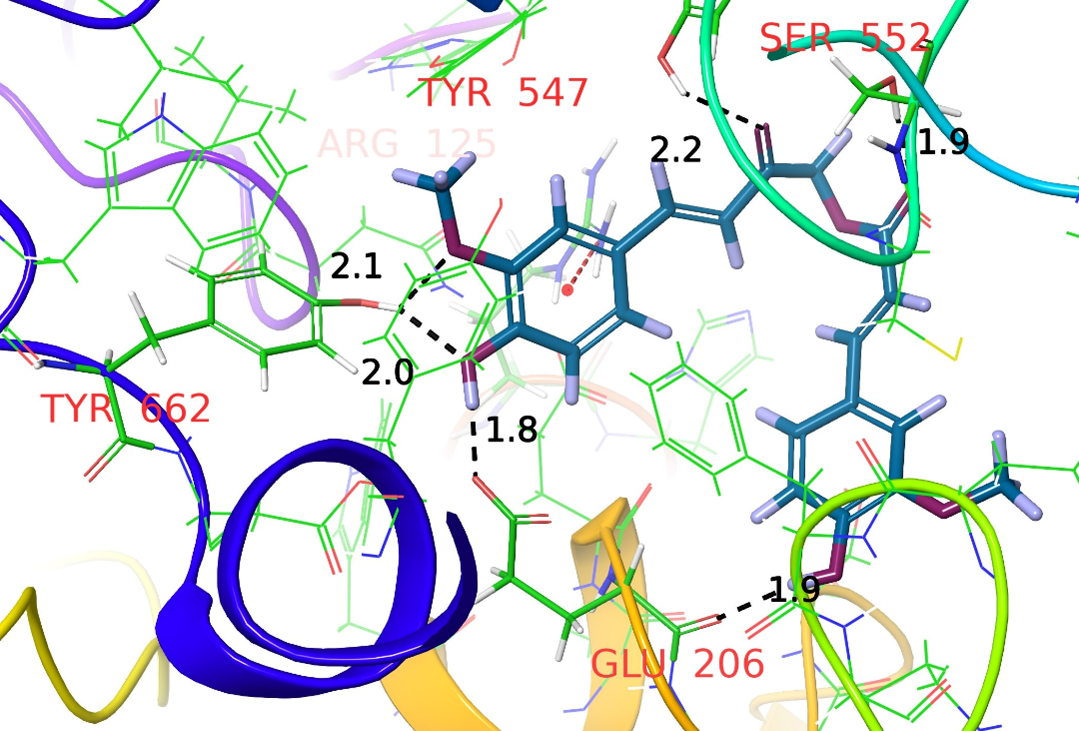


Figure S4: MD trajectory pose of CA in the active site of DPP-IV at 0ns


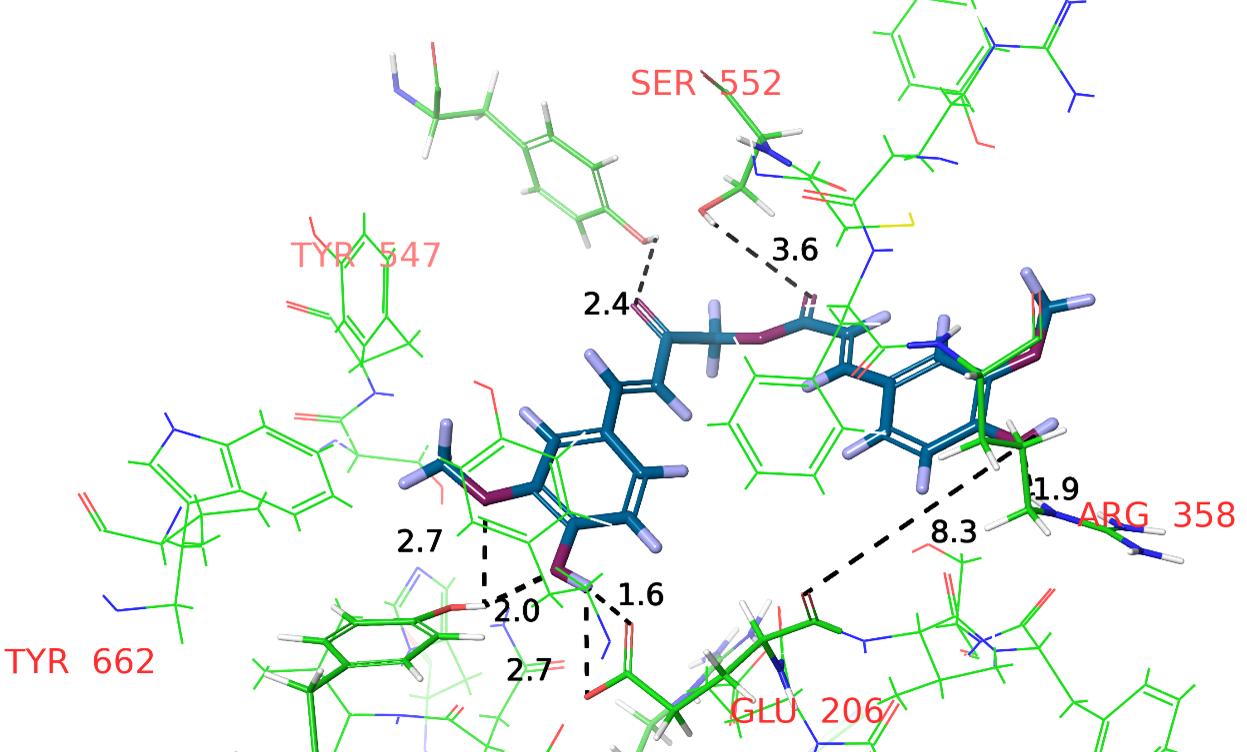


Figure S5: MD trajectory pose of CA in the active site of DPP-IV at 5ns


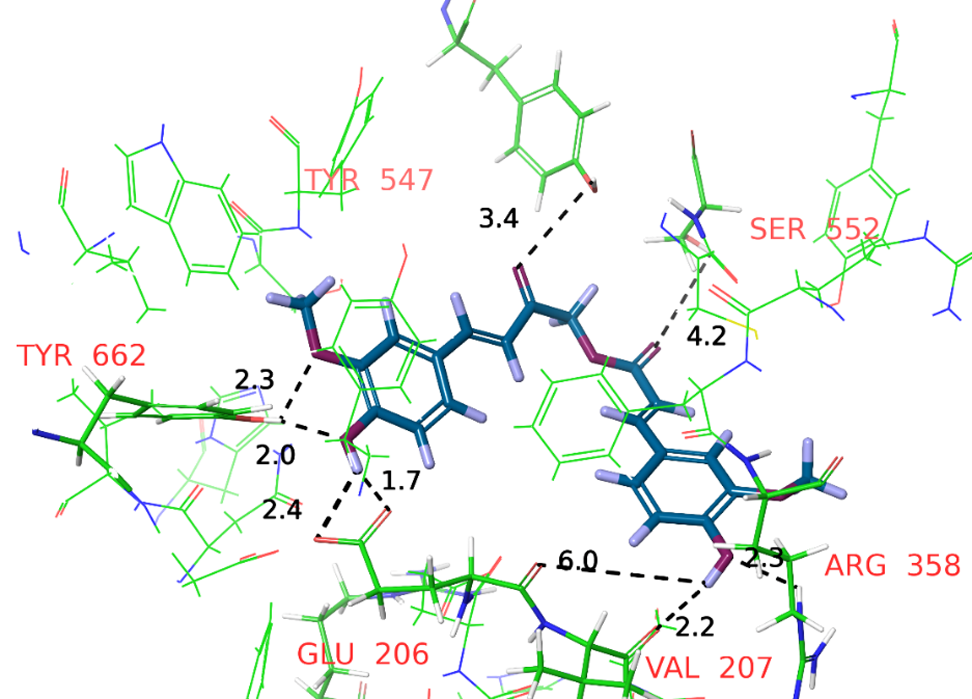


Figure S6: MD trajectory pose of CA in the active site of DPP-IV at 10ns


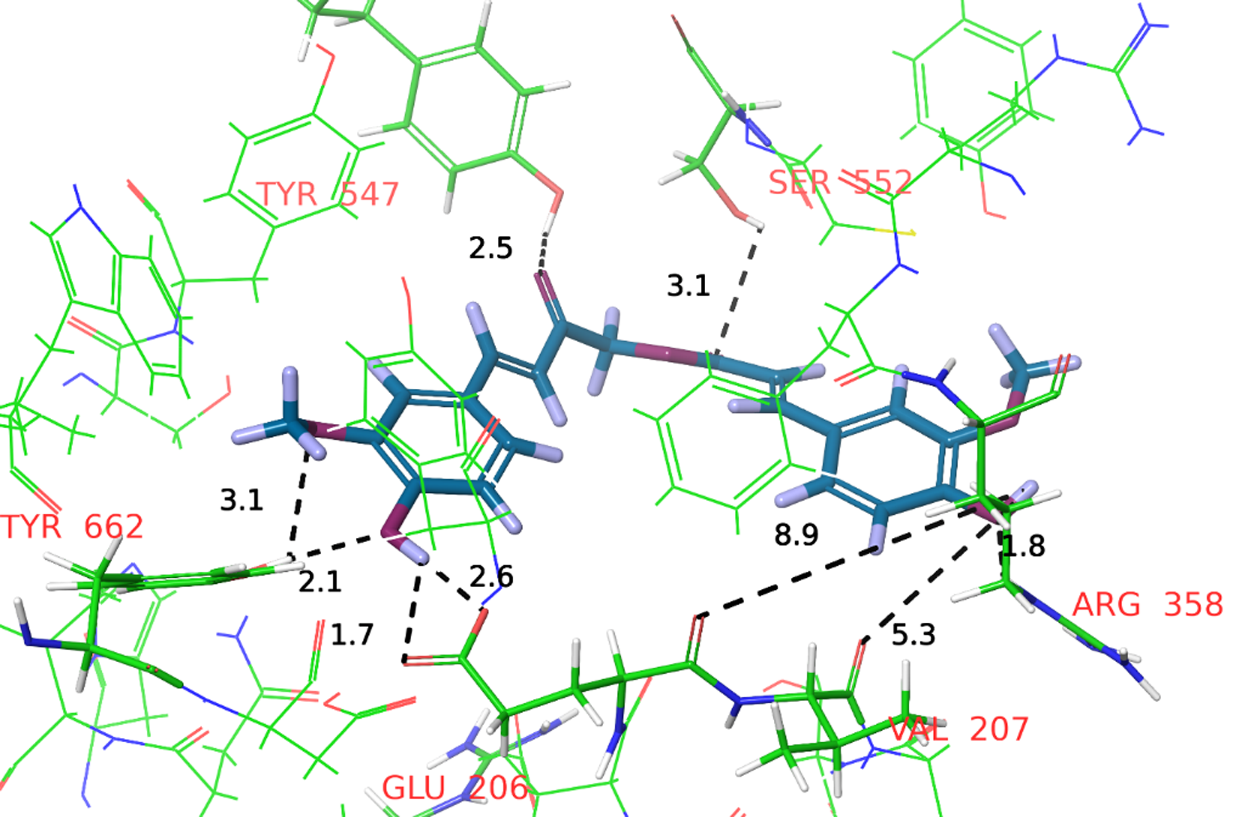


Figure S7: MD trajectory pose of CA in the active site of DPP-IV at 15ns


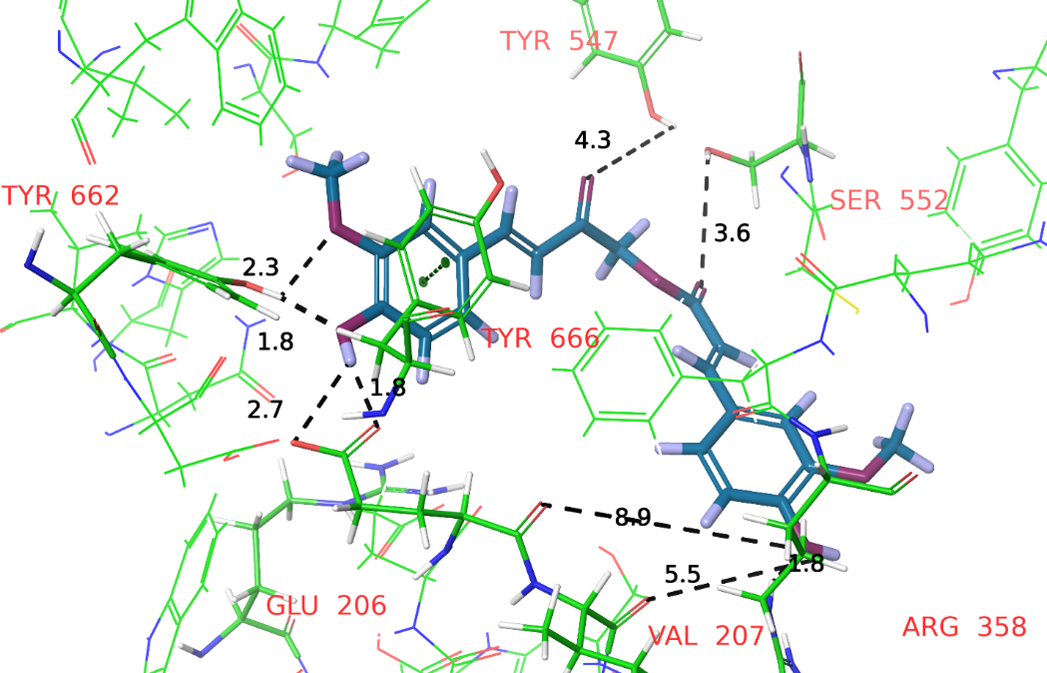


Figure S8: MD trajectory pose of CA in the active site of DPP-IV at 20ns


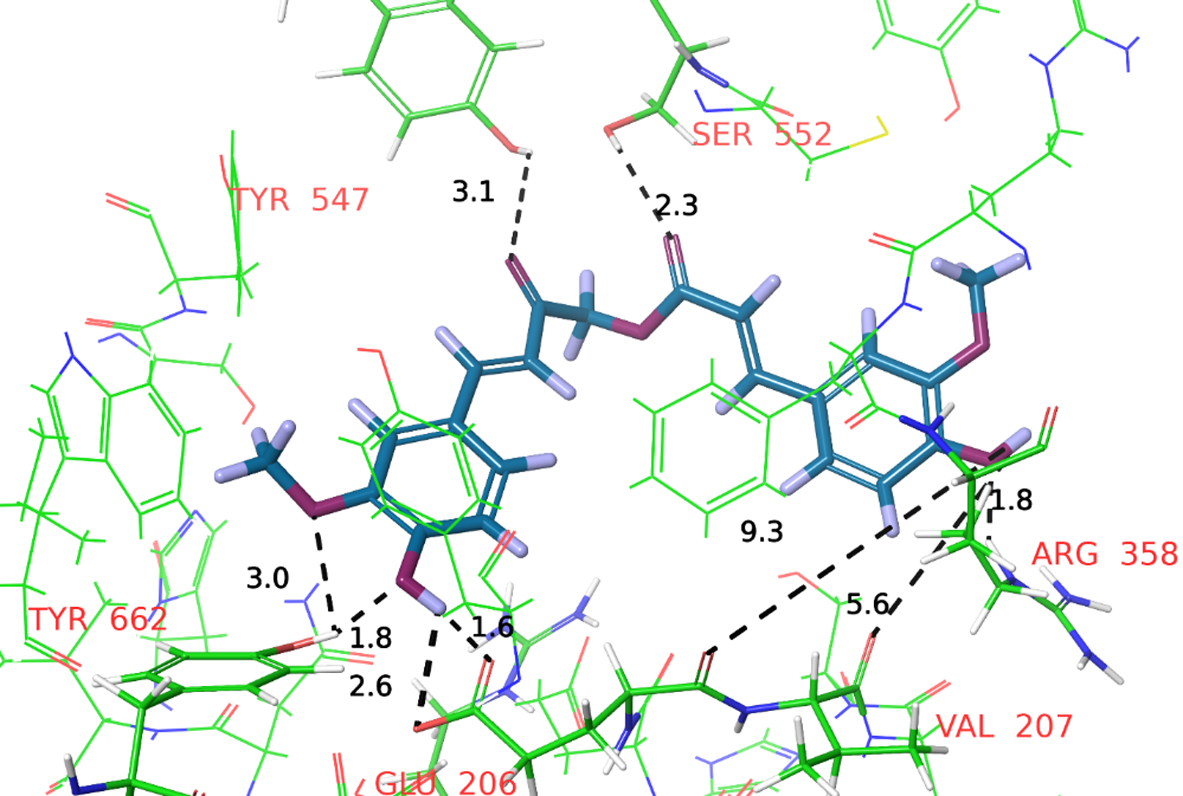


Figure S9: MD trajectory pose of CA in the active site of DPP-IV at 25ns


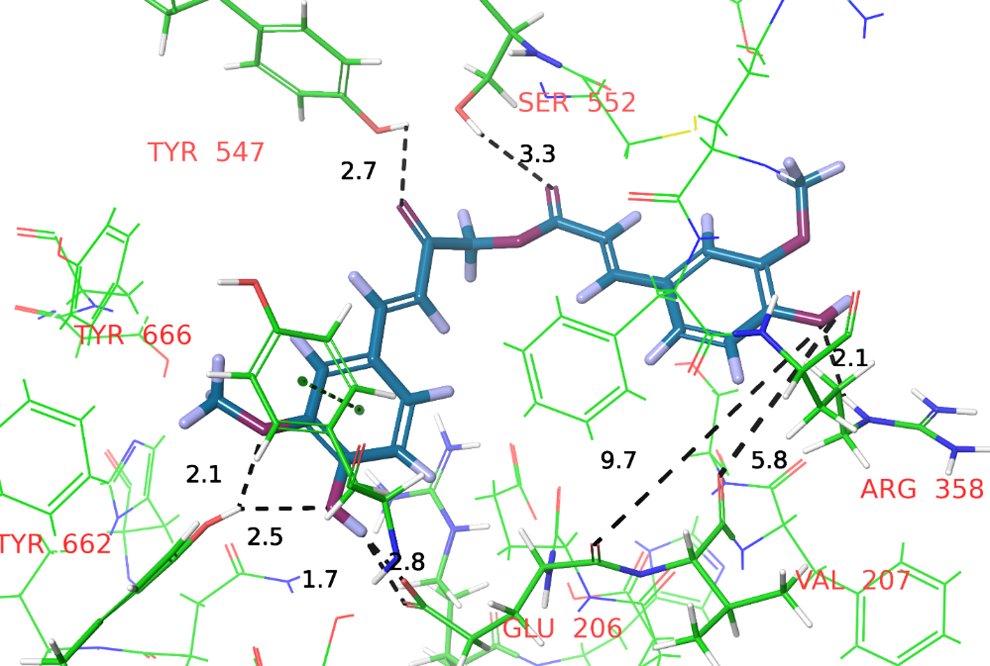


Figure S10: MD trajectory pose of CA in the active site of DPP-IV at 30ns


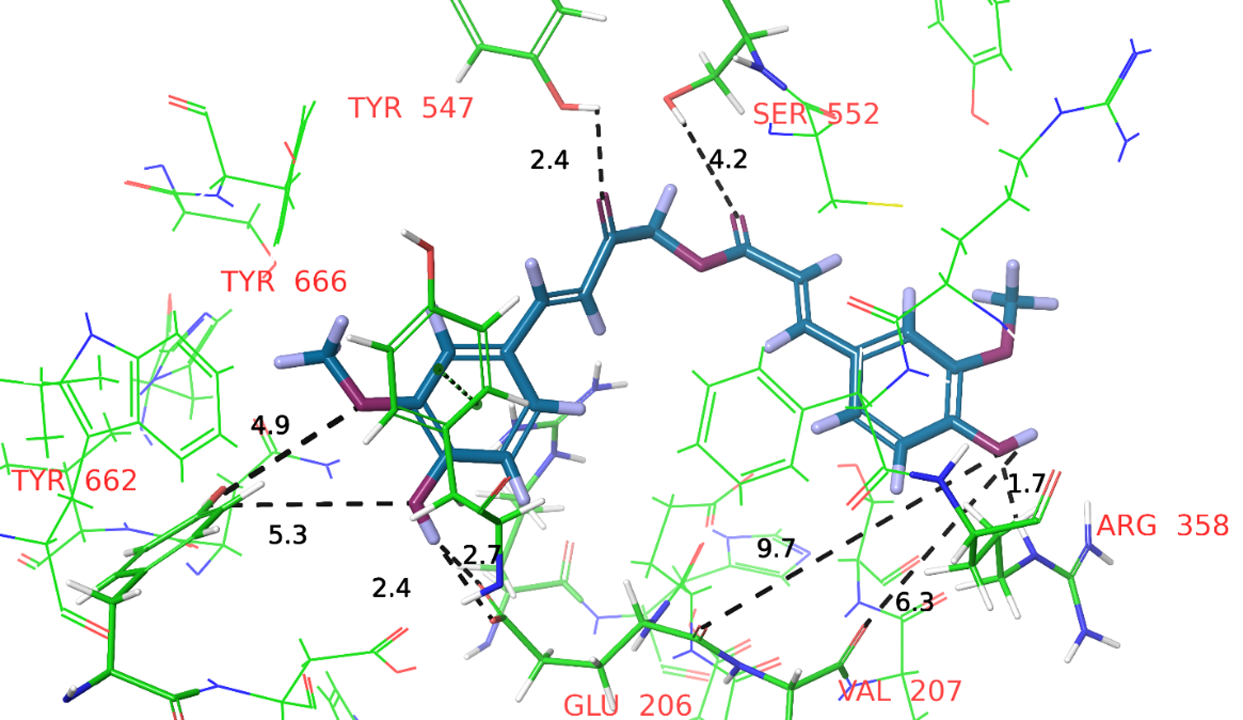


Figure S11: MD trajectory pose of CA in the active site of DPP-IV at 31ns


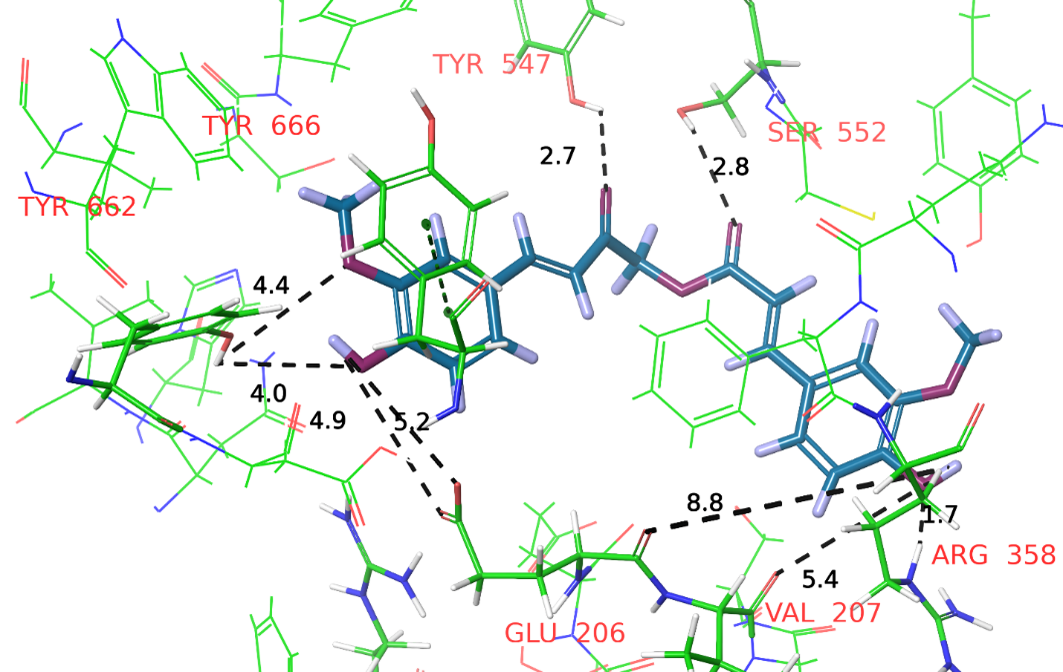


Figure S12: MD trajectory pose of CA in the active site of DPP-IV at 32ns


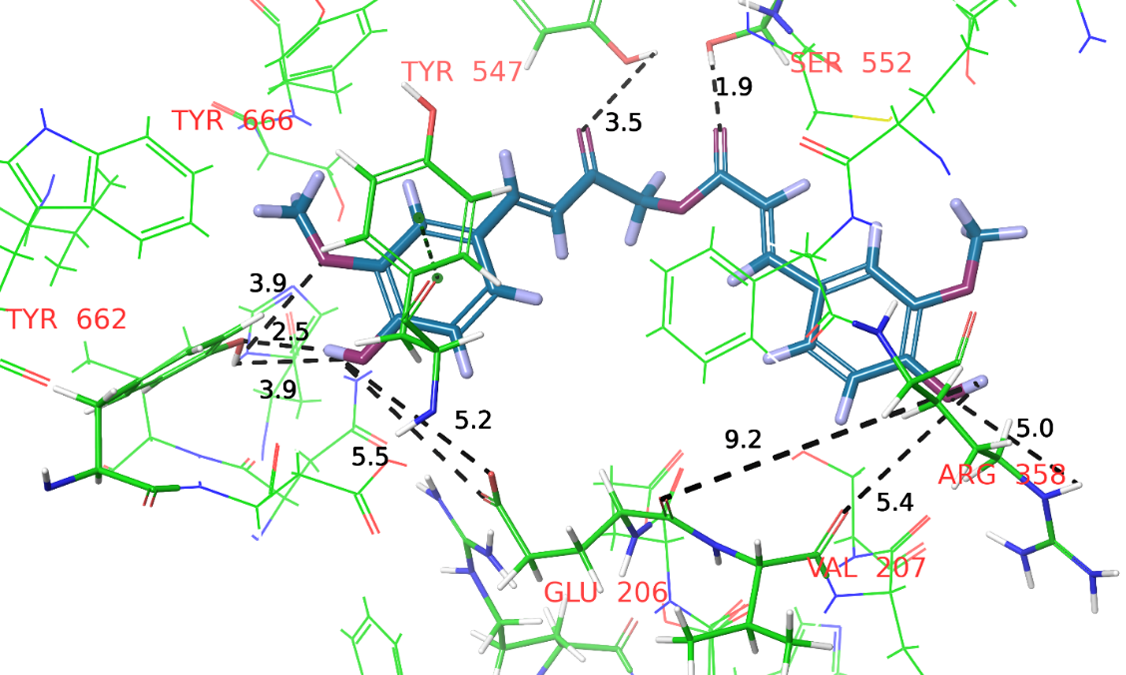


Figure S13: MD trajectory pose of CA in the active site of DPP-IV at 33ns


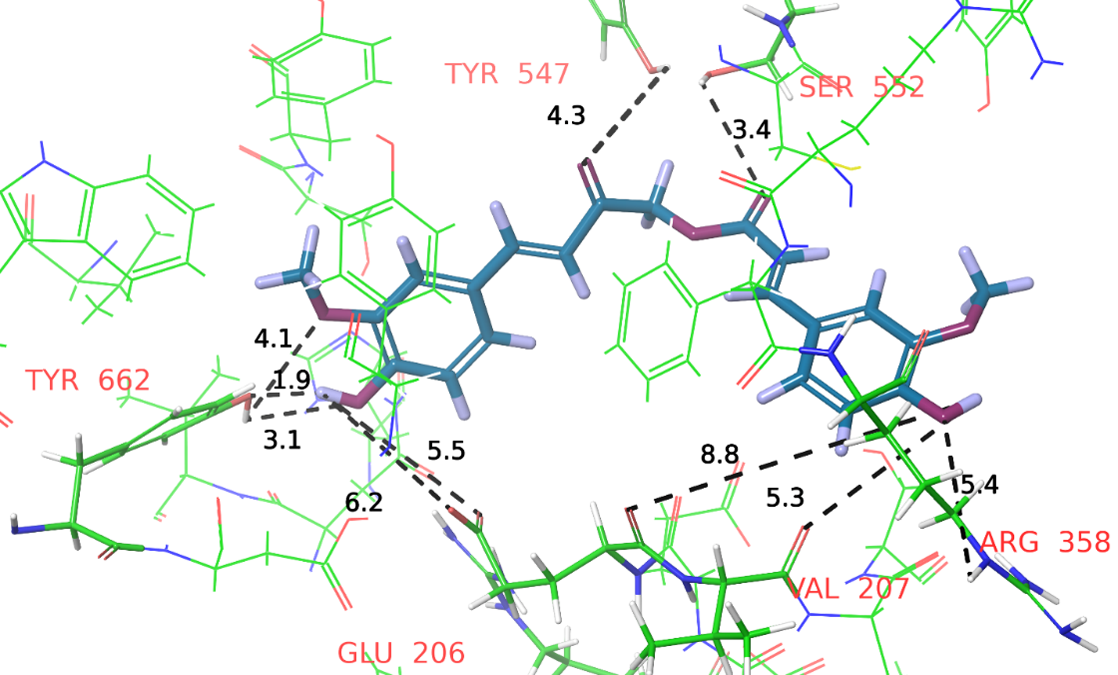


Figure S14: MD trajectory pose of CA in the active site of DPP-IV at 34ns


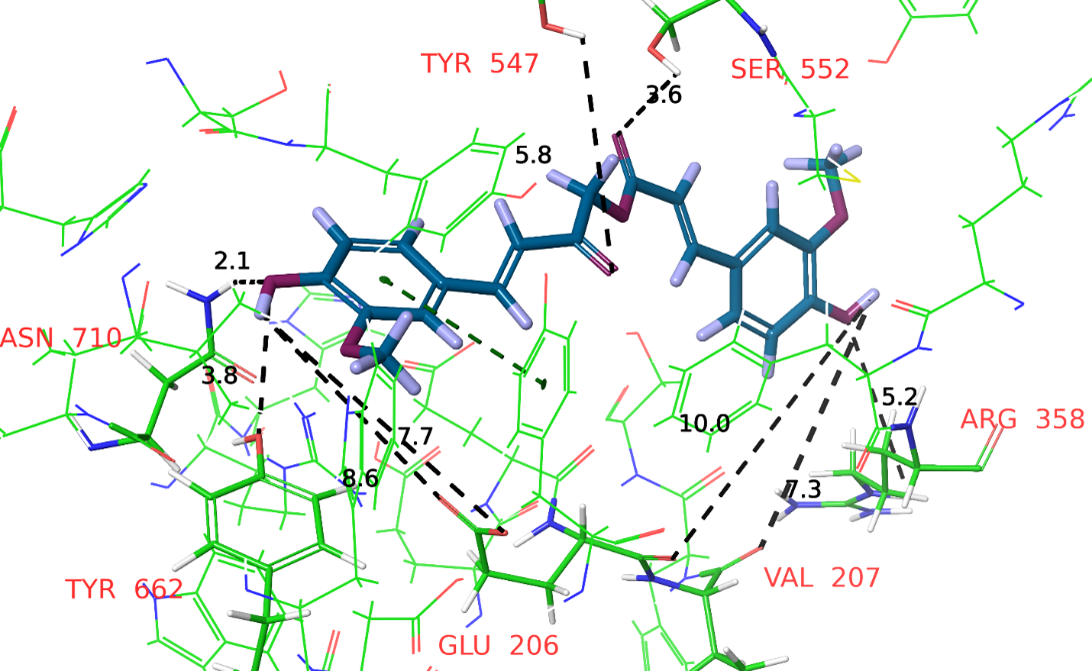


Figure S15: MD trajectory pose of CA in the active site of DPP-IV at 35ns


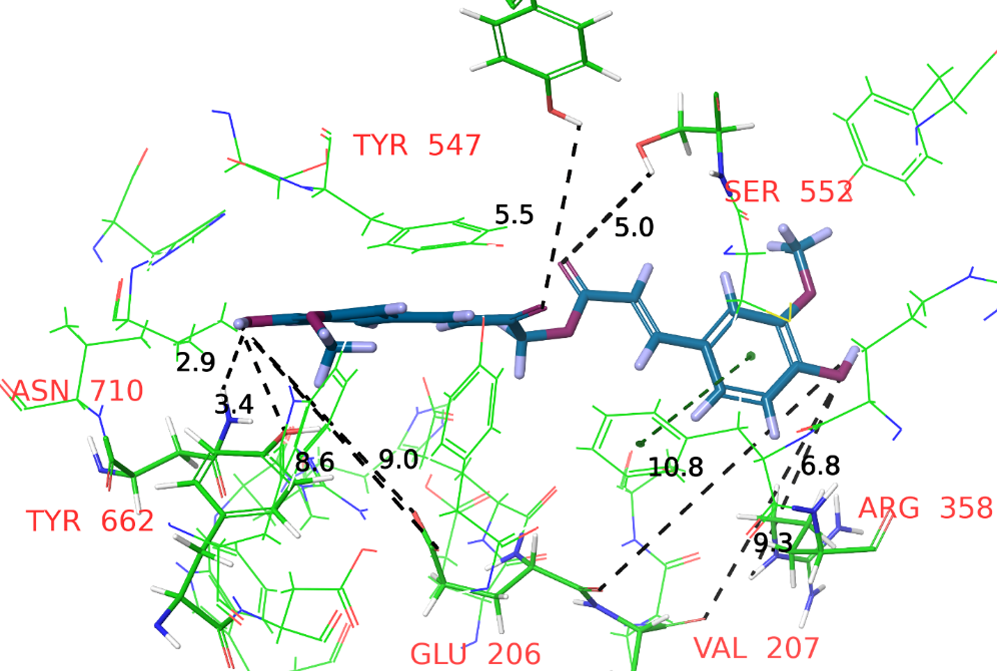


Figure S16: MD trajectory pose of CA in the active site of DPP-IV at 40ns


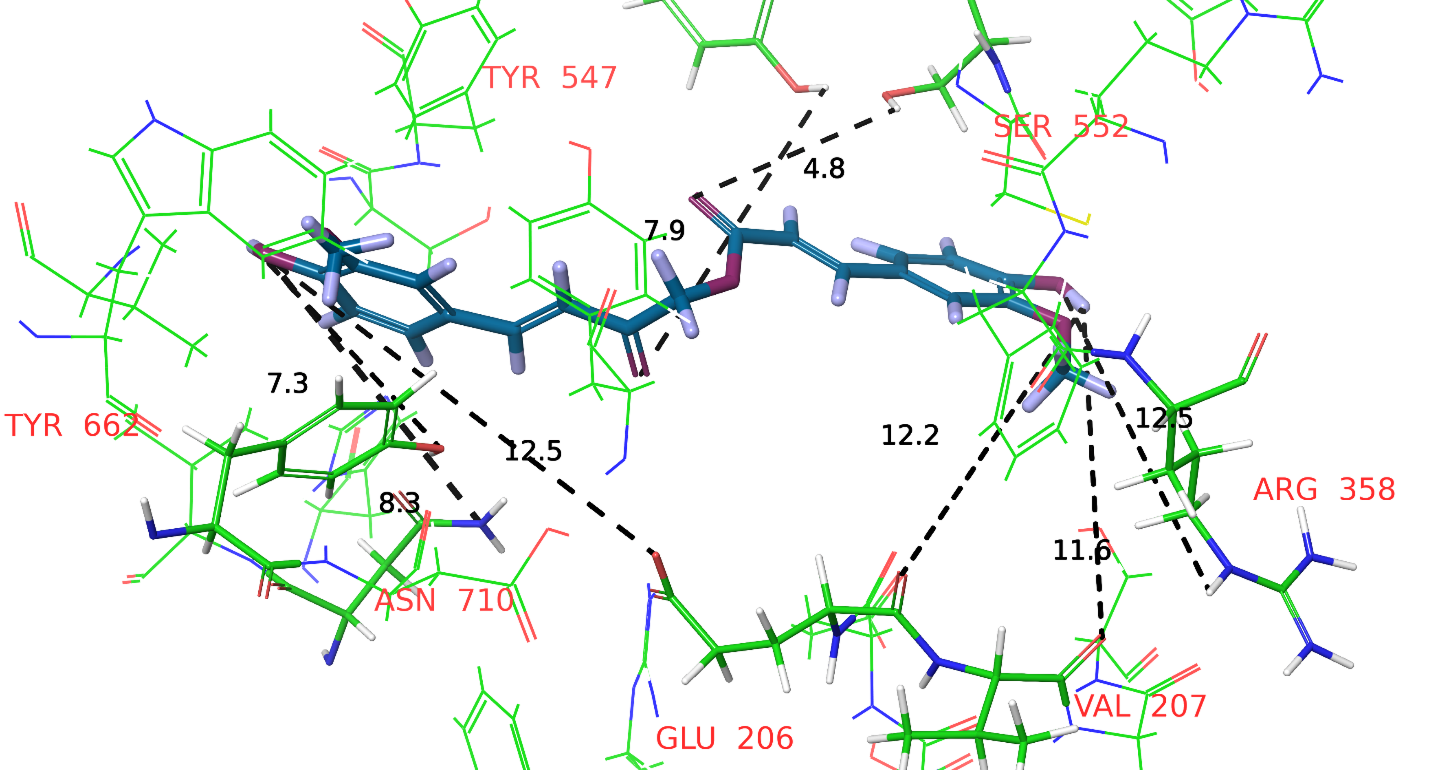


Figure S17: MD trajectory pose of CA in the active site of DPP-IV at 45ns


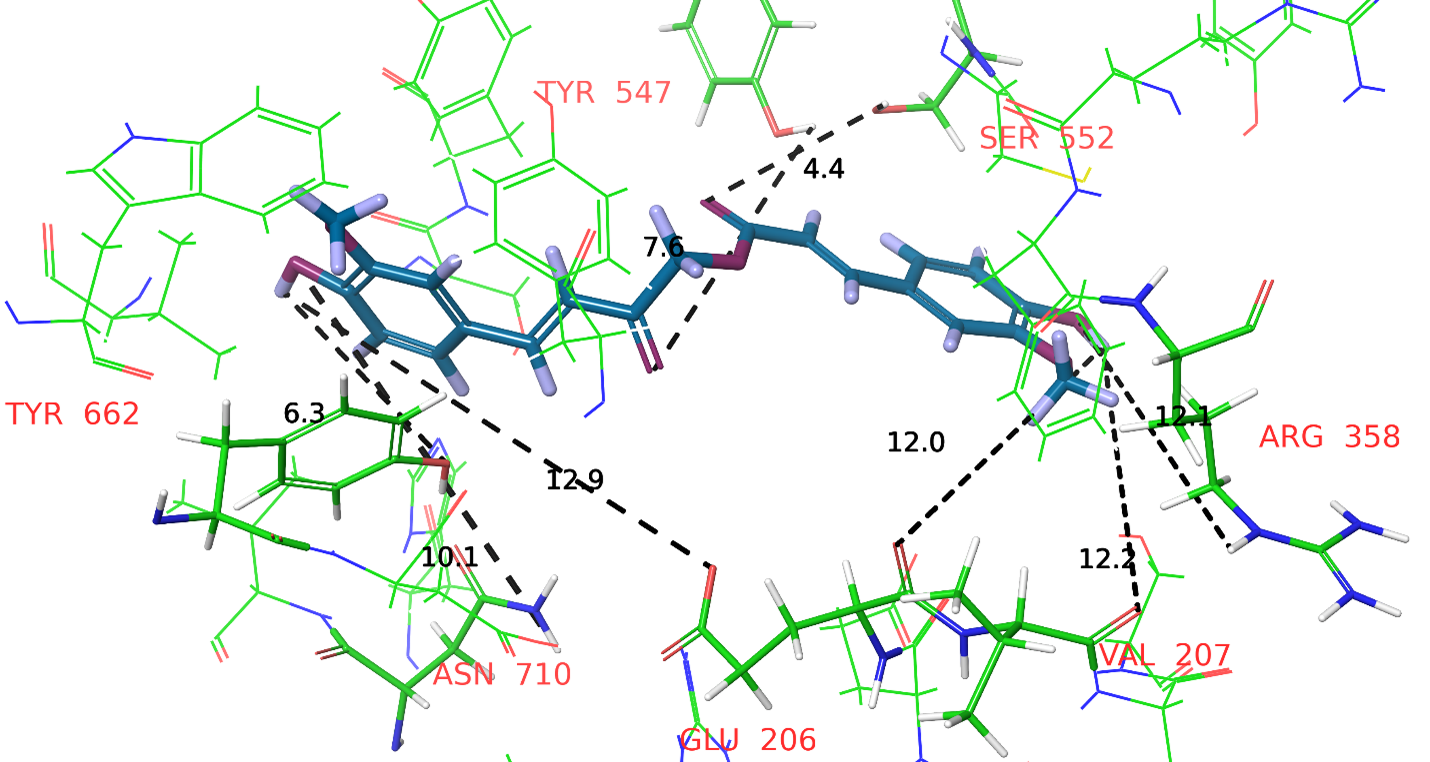


Figure S18: MD trajectory pose of CA in the active site of DPP-IV at 50ns


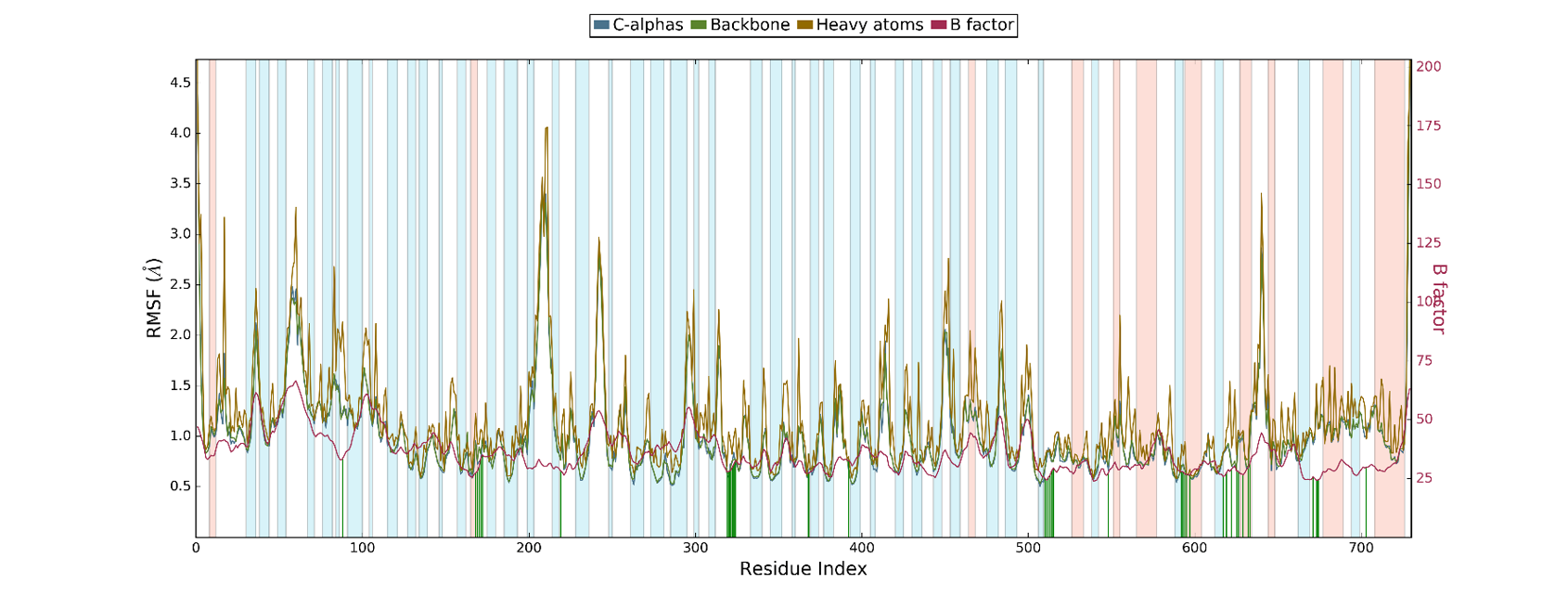


Figure S19: RMSF Plot and Protein –Ligand contacts of Calebin A


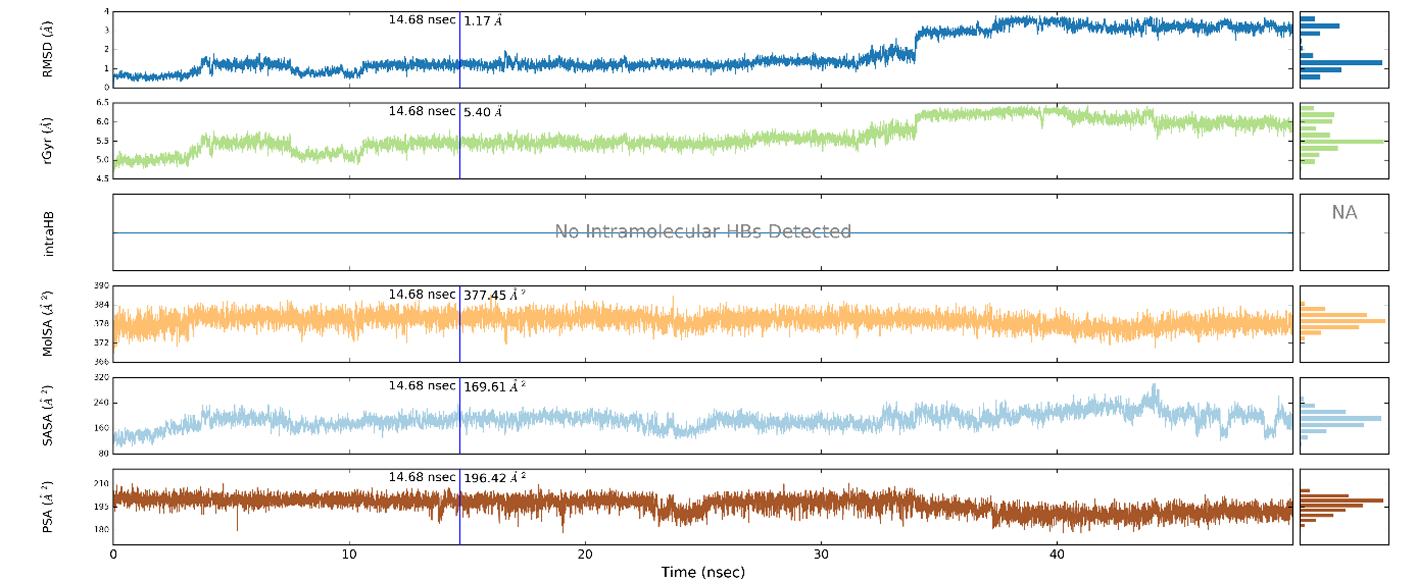


Figure S20: Root-mean-square deviation (RMSD), radius of gyration (rGyr), Molecular surface area (MolSA), Solvent Accessible Surface Area (SASA), and Polar Surface Area (PSA) plots of CA.


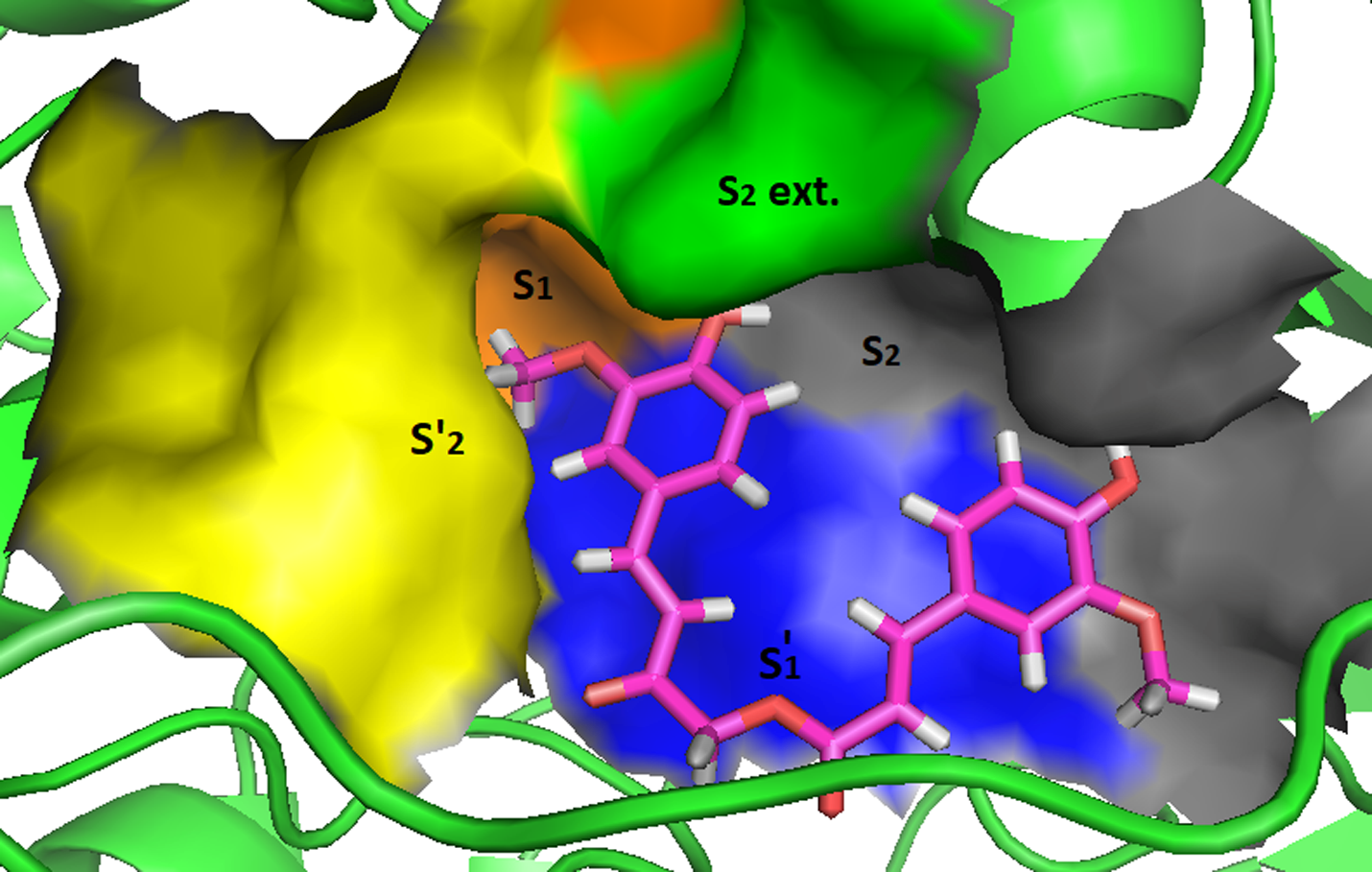


Figure S21: Binding mode of Calebin A in the active site of DPP-IV generated by PyMOL.


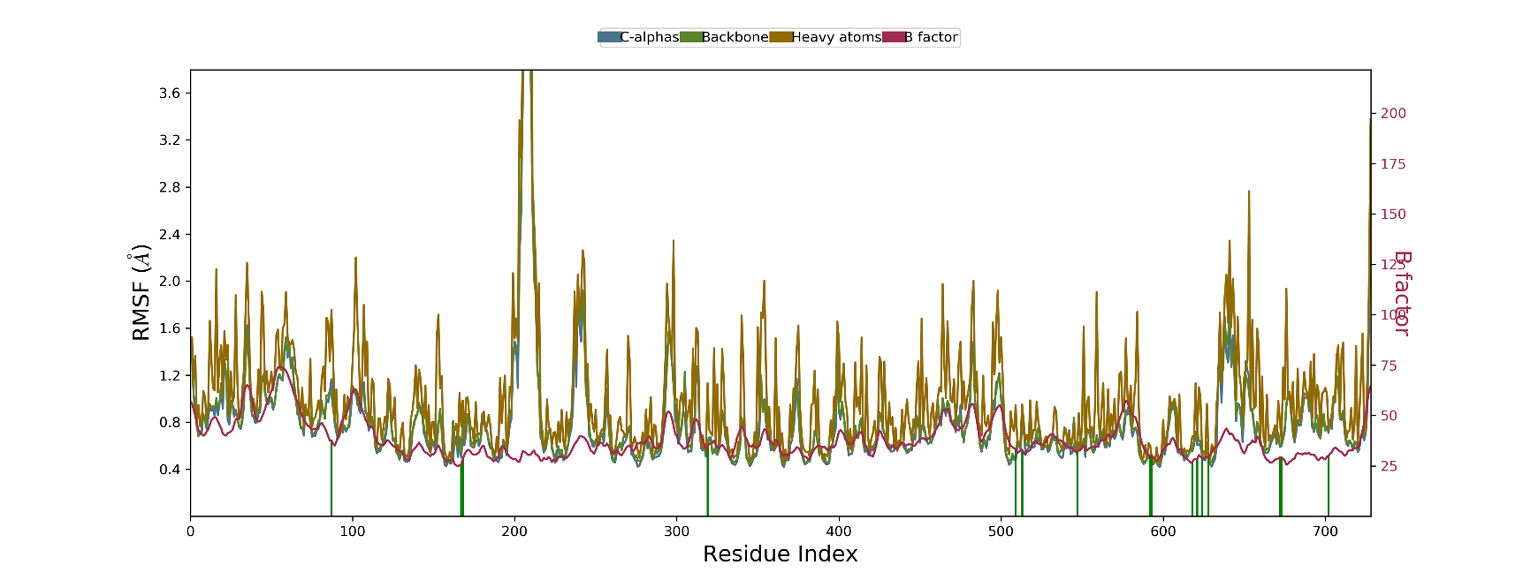


Figure S22: RMSF plot and ligand contacts of teneligliptin with DPP-IV (PDB ID: 3VJK) throughout the 50 ns simulation
